# Time series single-cell transcriptional atlases reveal cell fate differentiation driven by light in *Arabidopsis* seedlings

**DOI:** 10.1038/s41477-023-01544-4

**Published:** 2023-10-30

**Authors:** Xue Han, Yilin Zhang, Zhiying Lou, Jian Li, Zheng Wang, Chunlei Gao, Yi Liu, Zizheng Ren, Weimin Liu, Bosheng Li, Wenbo Pan, Huawei Zhang, Qing Sang, Miaomiao Wan, Hang He, Xing Wang Deng

**Affiliations:** 1 National Key Laboratory of Wheat Improvement, Peking University Institute of Advanced Agricultural Sciences, Shandong Laboratory of Advanced Agricultural Sciences at Weifang, Weifang, Shandong China; 2 School of Advanced Agricultural Sciences and School of Life Sciences, Tsinghua-Peking Center for Life Sciences, Peking University, Beijing, China

**Keywords:** Cell fate, Light responses, Plant morphogenesis

## Abstract

Light serves as the energy source for plants as well as a signal for growth and development during their whole life cycle. Seedling de-etiolation is the most dramatic manifestation of light-regulated plant development processes, as massive reprogramming of the plant transcriptome occurs at this time. Although several studies have reported about organ-specific development and expression induced by light, a systematic analysis of cell-type-specific differentiation and the associated transcriptional regulation is still lacking. Here we obtained single-cell transcriptional atlases for etiolated, de-etiolating and light-grown *Arabidopsis* *thaliana* seedlings. Informative cells from shoot and root tissues were grouped into 48 different cell clusters and finely annotated using multiple markers. With the determination of comprehensive developmental trajectories, we demonstrate light modulation of cell fate determination during guard cell specialization and vasculature development. Comparison of expression atlases between wild type and the *pifq* mutant indicates that phytochrome-interacting factors (PIFs) are involved in distinct developmental processes in endodermal and stomatal lineage cells via controlling cell-type-specific expression of target genes. These results provide information concerning the light signalling networks at the cell-type resolution, improving our understanding of how light regulates plant development at the cell-type and genome-wide levels. The obtained information could serve as a valuable resource for comprehensively investigating the molecular mechanism of cell development and differentiation in response to light.

## Abstract (web summary)

Using transcriptomic data from ~100,000 informative cells, this study constructed a dynamic cell atlas during the process of de-etiolation induced by light for *Arabidopsis* seedlings, revealing comprehensive development responses at single-cell resolution.

## Main

Cell fate specification and differentiation are core development processes in multicellular organisms and are regulated by intracellular molecular networks and extracellular environmental signals. Most plants that begin from seeds undergo a dramatic developmental switch from skotomorphogenesis (development patterns that occur under darkness, or etiolation) to photomorphogenesis (development that occurs under light) when seedlings emerge from the soil. When seeds first germinate in the soil, cell elongation in the hypocotyl is maximized to reach the light^1^. After 2 d of skotomorphogenesis, the development of cotyledons is essentially arrested in the dark^1^. However, when seedlings finally emerge from the soil, different development processes in distinct shoot cell types proceed forward to facilitate photosynthesis; these processes include cotyledon cell expansion and development^2,3^, stomatal differentiation^4,5^ and chloroplast development^6^. However, the developmental patterns of roots are not dramatically affected by light during de-etiolation^1^.

Although the exact patterns of morphogenesis vary widely among different taxa, the core light signalling machinery is functionally conserved from single-celled algae to angiosperms^7–14^. In the past 30 years, comprehensive signalling networks underlying how light controls *Arabidopsis* seedling development have been constructed^15–17^; however, most of these networks have been analysed at the whole-seedling or organ level. Organ-specific light control of genome expression has been reported decades ago^18^. At the transcriptional level, light-responsive genes show distinct expression patterns in the cotyledons, hypocotyls and roots^18–21^. Intriguingly, tissue-specific photoreceptors^22,23^ and core repressors^24^ have been shown to exert different abilities to rescue mutant phenotypes. Compared with other tissues, the vascular system plays more critical roles in plant responses to light, as vascular-specific expression of one photoreceptor^23^ and one light signalling factor^24^ enabled corresponding mutants to regain wild-type (WT) phenotypes. Furthermore, light can induce a reboot of cell development and differentiation, which is arrested during skotomorphogenesis. In particular, stomatal cells were shown to be abnormal under darkness and to have a lower density, and many were retained as precursors^4^. Light enhances the development process and ensures the proper patterning and opening of guard cells (GCs). In addition, vasculature differentiation is repressed under darkness compared with constant light^25^. These are considered intrinsic cell-type-specific signalling and developmental processes. However, light-regulated cell-type-specific transcriptional and developmental responses have been largely unexplored in previous studies because of technical bottlenecks^20,21^.

In this study, we carried out time-series single-cell RNA sequencing (scRNA-seq) analyses of the shoots and roots of de-etiolated *Arabidopsis* seedlings and constructed a spatiotemporal cell atlas comprising 92,861 valid cells. The shoot cells displayed gradual but dramatic changes in transcriptional states; however, the root cells were quite stable in the presence and absence of light. After annotating and validating cell types, we identified 12,447 cell cluster preferentially expressed genes and 73 spatiotemporal expression modules. With information on both cell type and light duration, we estimated the developmental dynamics of each shoot cell type in the context of de-etiolation, enabling us to identify respective cell differentiation trajectories under respective light conditions and to identify the novel regulators involved. To reveal the cell-type-specific mechanisms modulated by light signalling networks, extensive comparisons of cell atlases of the *pifq* mutant and WT were carried out. Collectively, our atlases and findings improve our understanding of the heterogeneity of light responses at cell-type resolution, facilitating studies of light signalling and cell development.

## Results

### Construction of de-etiolating seedling cell atlases

When dark-grown *Arabidopsis* seedlings are exposed to light, a series of dramatic changes occur, including cotyledon expansion and greening and apical hook opening. To illustrate transcriptional transitions in distinct cell types, shoot and root tissues of *Arabidopsis* seedlings that were cultivated under constant darkness, were de-etiolating (exposed to light for 1 h, 6 h and 24 h) or were grown under constant light were collected for scRNA-seq (Methods, and Supplementary Figs. 1 and 2). Bulk RNA-seq data for corresponding samples were also obtained for cotyledon, hypocotyl and root (Supplementary Fig. 3). Tissues for scRNA-seq were dissected for cell wall digestion, and the dissociated protoplasts were separately loaded into a 10x Genomics Chromium Controller. Subsequently, the barcoded molecules were amplified and sequenced via an Illumina Nova-Seq platform. Cells with extreme values of total unique molecule identifier (UMI) counts and the organelle molecule content were discarded. As a result, a total of 31,796 and 61,065 informative cells from shoot and root samples, respectively, were profiled for the construction of the final transcriptomic atlas (Supplementary Tables 1–3 and Supplementary Fig. 4a,b).

**Fig. 1:**
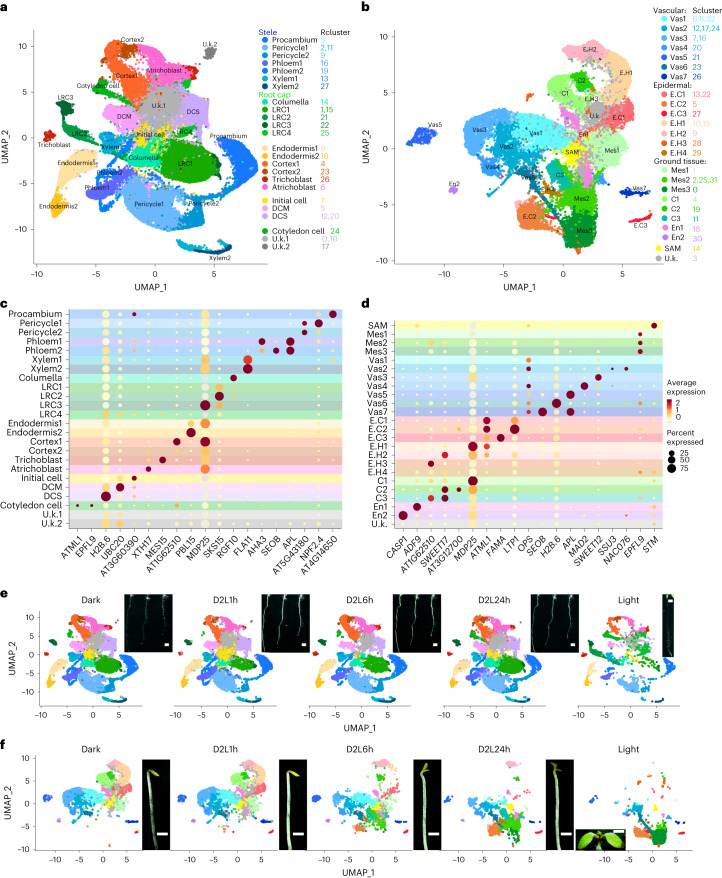
Cell atlases of de-etiolating seedlings. **a**, Visualization of root cell types (states) via UMAP. The dots indicate individual cells, while the colours represent the respective cell types. Corresponding root cluster (rcluster) IDs are indicated on the right. **b**, Visualization of shoot cell types (states) via UMAP. The dots indicate individual cells, while the colours represent the respective cell types. Corresponding shoot cluster (scluster) IDs are indicated on the right. **c**, Expression patterns of representative marker genes for root cell types. The dot diameter indicates the proportion of cluster cells expressing a given gene and the colour indicates relative expression levels. **d**, Expression patterns of representative marker genes for shoot cell types. The dot diameter indicates the proportion of cluster cells expressing a given gene and the colour indicates relative expression levels. **e**, Time-series root cell atlas and phenotypes in different light radiation timepoints. Two replicates of Dark samples were merged. **f**, Time-series shoot cell atlas and phenotypes in different light radiation timepoints. Two replicates of Dark samples were merged.

Root (Fig. 1a) and shoot (Fig. 1b) cells were used for reconstruction of transcriptome atlases (Methods) (Fig. 1), and classified into 28 root clusters (rcluster0–27) (Supplementary Fig. 4c) and 33 shoot clusters (scluster0–32) (Supplementary Fig. 4d). Previous *Arabidopsis* cell atlases^26–37^ facilitated fast and accurate cell type annotation. Taking advantage of the expression patterns of these known markers (Fig. 1c,d, Supplementary Table 4 and Supplementary Figs. 5 and 6), we ultimately annotated most root and shoot cell clusters. A more comprehensive interpretation of the dynamic atlases could be obtained by comparing among timepoints. As illustrated, the states of root cell types were stable (Fig. 1e), but extensive changes could be observed for shoot cell types (Fig. 1f). Intriguingly, distinct shoot cell types underwent different degrees of developmental changes during de-etiolation. Mesophyll (Mes), epidermis (E) and cortex (C) cells experienced gradual but radical changes in transcriptional status (the location of cell types changed from upper to lower parts on the uniform manifold approximation and projection (UMAP)); however, the vascular cell clusters (Vas) were relatively insensitive to light (the location of Vas cells on the UMAP was almost unchanged) (Fig. 1f). Therefore, we focused on shoot cell types in subsequent profiles.

**Fig. 2:**
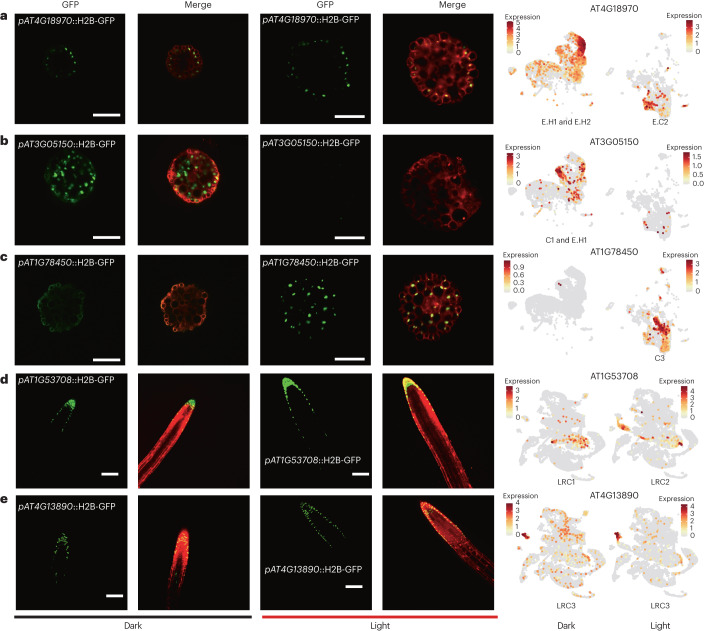
Specific expression of marker genes for hypocotyl cell types and lateral root caps. **a**, Expression of *AT4G18970* in hypocotyl epidermal cells under dark and light conditions. The gene expression pattern was determined by *AT4G18970* promoter-driven Histone2B-GFP (H2B-GFP, green) reporter. The cell outline (red) was visualized by FM4-64 staining in addition to DsRed2 reporter driven by a seedling-specific promoter (*pAT2S3*). **b**, Expression of *AT3G05150* in hypocotyl cortical cells specifically in darkness. The gene expression pattern was determined using Histone2B-GFP (H2B-GFP, green) reporter driven by *AT3G05150* promoter. The cell outline (red) was visualized by FM4-64 staining in addition to DsRed2 reporter driven by a seedling-specific promoter (*pAT2S3*). **c**, Expression of *AT1G78450* in hypocotyl cortical cells specifically in light conditions. The gene expression pattern was determined by *AT1G78450* promoter-driven Histone2B-GFP (H2B-GFP, green) reporter. The cell outline (red) was visualized by FM4-64 staining in addition to DsRed2 reporter driven by a seedling-specific promoter (*pAT2S3*). **d**, Expression of *AT1G53708* in subtype LRC1 and LRC2 in dark and light conditions, respectively. The seedling cells (red) were labelled with the seedling-specific promoter *AT2S3*::DsRed2. **e**, Expression of *AT4G13890* in a stable subtype LRC3 in dark and light conditions. The seedling cells (red) were labelled with the seedling-specific promoter *AT2S3*::DsRed2. The expression patterns of marker genes in cell atlases for Dark and Light samples are correspondingly illustrated on the right. Colour bar, normalized UMI counts; darker colours indicated higher expression. Scale bar, 100 μm. Each experiment was independently repeated three times.

As available markers for hypocotyl cell types and subtypes of lateral root cap (LRC) are lacking, promoter-reporter analyses for five highly expressed genes were carried out (Fig. 2). The expression of *AT4G18970* in transverse sections of hypocotyls verified the hypocotyl epidermal clusters on the atlas (Fig. 2a). The promoter signal of *AT3G05150* (Fig. 2b) and *AT1G78450* (Fig. 2c) revealed the cortical identity for cell clusters in dark and light, respectively. Different cell clusters belonging to LRC were also verified by specific promoter signals of *AT1G53708* (Fig. 2d) and *AT4G13890* (Fig. 2e), indicating transcriptomic pattern changes in LRC between growth in constant-dark and constant-light conditions. With the aid of known markers and reporter lines, the finely annotated high-resolution atlases were generated.

### Identification of spatiotemporal markers

**Fig. 3:**
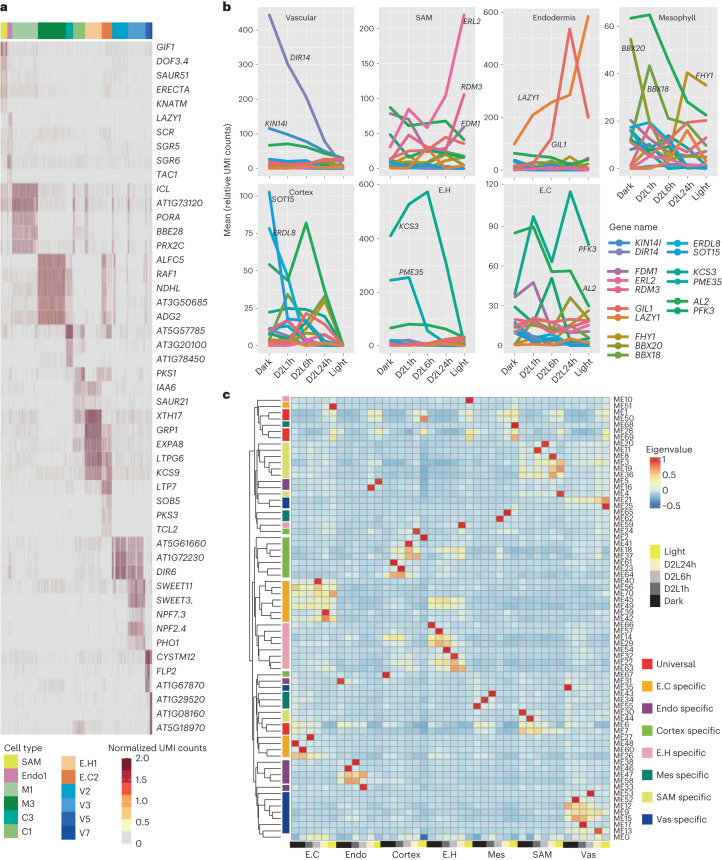
Expression of spatiotemporal-specific genes. **a**, Expression of the spatio-markers in each cell; each column represents a cell. The annotated colours for the columns indicate the respective cell types. **b**, Dynamic expression of spatio-markers during de-etiolation. **c**, Spatiotemporal co-expression networks for seven shoot cell types during de-etiolation. The annotated colours of the rows represent different patterns of co-expression modules and the annotated colours for columns represent de-etiolation times.

Characterizing spatiotemporally specifically expressed genes in individual cell types and at different development stages can be used as a powerful tool for elucidating intrinsic molecular regulatory modules in multicellular organisms. Organ-specific genes in response to light^20,21^ and cell type makers^27,37^ have been identified separately. However, parsing expression dynamics of light-responsive genes in cell-type resolution is unclear. We identified 9,997 and 6,702 genes with obvious expression preference to specific root and shoot cell types, respectively (Supplementary Tables 5 and 6, and Methods). As expected, a batch of known spatial markers was included in the list (Figs. 1b,c and 3a). For instance, genes responsive to gravity, such as *LAZY1*, *LAZY2*, *LAZY4* (refs. ^37,38^), *TAC1* (ref. ^38^), *SGR5*, *SGR6* (refs. ^39,40^) and *SCR*^4^, were identified on the basis of their specific expression in endodermal cells (En1 and En2). In addition, a number of cell type-specific transporters, including *SWEET11* (ref. ^41^), *SWEET3* (*AT5G53190*), *NPF7.3* (ref. ^42^) and *NPF2.4* (ref. ^43^), were highly expressed in vascular parenchymal cells (Fig. 3a).

Notably, clusters in the atlases were classified by thousands of features (highly variable genes), reflecting the variance not only due to spatial differences but also due to the diversity of light exposure times. To classify the light-regulated genes from among all the cluster-specific genes, we first processed cotyledon, hypocotyl and root tissues from the seedlings during dark-to-light transition for bulk transcriptome sequencing (Methods and Supplementary Fig. 2). Using organ-specific RNA-seq data of de-etiolated seedlings in this study and previously published ones, we identified 13,653 genes that were significantly differentially expressed in at least one organ at one light exposure timepoint compared with that under darkness (Methods and Supplementary Table 7). More than 60% of the light-induced differentially expressed genes (DEGs) (8,212/13,653) were identified as being differentially expressed among cell types and constituted 66.0% (8,212/12,447) of DEGs identified with the scRNA-seq data. Notably, many spatial markers could be regulated by light (Fig. 3b). Identifying universal and cell-type-specific light-responsive genes would facilitate our understanding of cell development induced by light.

To extensively resolve gene spatiotemporal expression patterns, we constructed co-expression networks for seven shoot cell types: Mes, shoot apical meristem (SAM), En, Vas, C, E.cotydelon (E.C) and E.hypocotyl (E.H), at five different timepoints (Methods). The mean expression of each gene in cell types at each timepoint was calculated and further processed through a weighted gene co-expression network analysis (WGCNA) pipeline^44^. After filtration, a total of 21,543 qualified genes were clustered into 73 modules (Fig. 3c and Supplementary Table 8). The largest module, ME1, contained more than 12% (2,675) of the genes, the expression of which was universally induced by light in all the cell types studied. As expected, genes in ME1 were enriched in photosynthesis-related gene ontology (GO) terms (Supplementary Table 9). In contrast to the light-induced module ME1, genes from ME6 and ME7 were detected for higher expression in Dark samples without spatial preference. The photomorphogenesis-positive transcription factors *HY5*/*HYH* and negative transcription factors *PIF1* and *PIF5* were classified as belonging to ME1. Almost one-third of the direct target genes of HY5 (ref. ^20^) (80/295) were assigned to ME1 during de-etiolation. The target genes of PIFs^45,46^ (PIF1, PIF3, PIF4, PIF5) were enriched in both ME1 (43/333) and ME6 (30/333), and others were identified with spatial differences (Supplementary Table 8). In summary, basic light signalling networks were commonly induced in different cell types. However, regulated genes tended to be transcribed with a cell-type preference (Fig. 3b,c).

### Light balances the development of xylem and phloem

**Fig. 4:**
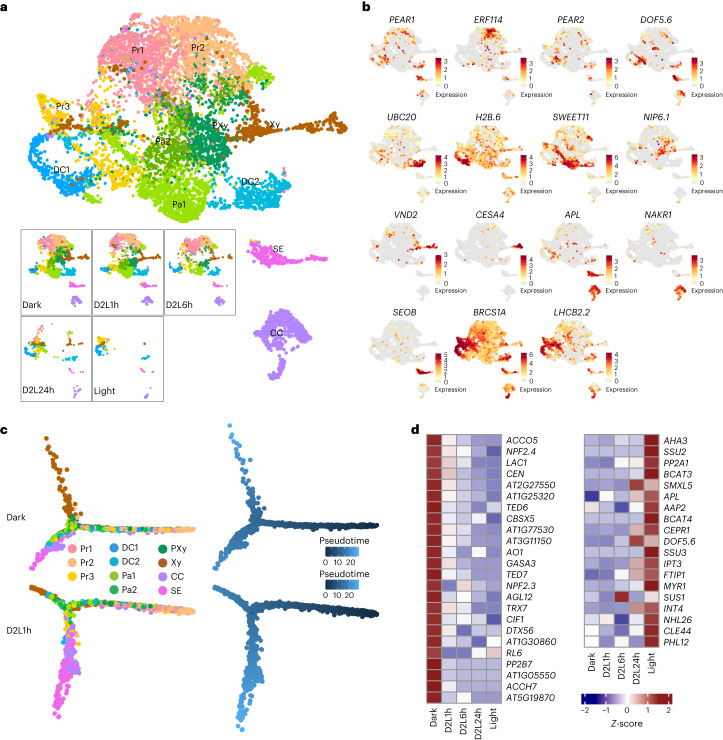
Light promotes phloem development but inhibits xylem development. **a**, Visualization of vascular cells via UMAP during the de-etiolation process for merged data (top) and respective data of different radiation times (bottom). **b**, Transcript distribution of subtype markers. Colour bar, normalized UMI counts. **c**, Visualization of vascular system development along with pseudotime. **d**, Expression trends of candidate genes involved in light-induced vasculature development. Pr, procambium; DC, dividing Cell; Pa, parenchyma; PXy, protoxylem; Xy, xylem; SE, phloem sieve element; CC, phloem companion cell.

The vasculature plays a vital role in water and solute transport, linking the development of shoots and roots. Compared with the outer layers of cells of shoot, the vascular system is quite stable, with similar cell-type composition and developmental processes (Figs. 1a and  4a). To shed light on the impact of de-etiolation on the vasculature, we screened 9,088 cells from vascular cell clusters (Vas1–Vas7) and re-clustered them according to vascular highly variable genes (Fig. 4a). Procambial cells (Vas1) were further classified into two subtypes (Pr1 and Pr2). The high expression of *PEAR1* and *PEAR2* (ref. ^47^) in these clusters verified their procambium identities (Fig. 4b). Except for the Vas1 (Pr1 and Pr2) clusters, procambial markers, including *DOF5.6* (ref. ^48^), were also expressed in scluster 17 (Fig. 4b, Supplementary Fig. 4d and Supplementary Table 5), which was annotated as Pr3. Procambial cells contain pluripotent stem cells and maintain their cell division ability to continually form new tissues^49^. Genes expressed in proliferating cells, such as *DOFs* and *ERF114*, were vigorously transcribed in procambial cell clusters (Pr1, Pr2 and Pr3) (Fig. 4b). Although the states of vasculature cells were relatively stable, the cell type composition of procambial clusters differed greatly during de-etiolation. Pr2 comprised the main type of procambial cells under darkness, and the Pr1 proportion increased after 1 h of light. Finally, Pr3 constituted the main position after 1 d of illumination (Fig. 4a). Furthermore, the proportion of dividing cells increased after light radiation (Supplementary Fig. 7). Taken together, procambial cells were at the centre of light sensing and signalling in the shoot vasculature system.

Xylem and phloem cells developed under both dark and light conditions (Fig. 4c). The development trajectories of vascular cells revealed two distinct development directions from procambial cells: the first trajectory is towards xylem cells and the other is towards phloem cells (Fig. 4c). To circumvent variances induced by large differences in cell numbers obtained between shoot samples, we only compared the developmental changes between Dark and Dark to Light (D2L)1h. The phloem branch had denser cells after 1 h of light exposure (Fig. 4c). To comprehensively study responsive genes specific in vascular cells, gene modules with higher eigenvalues in vasculature of the co-expression networks were screened out (Fig. 3c). There were 119 genes in ME52 (Supplementary Table 8), which were induced only in vascular cells of dark-grown seedlings, including a series of xylem development regulators. Among them, both TED6 and TED7 are type I membrane proteins with high sequence similarity and might function as components of a secondary cell wall cellulose synthase complex^50^. RNA interference (RNAi) of *TED6* or *TED7* could reduce the formation of tracheary elements^50^. NPF2.3 (ref. ^51^) and NPF2.4 (ref. ^43^) are two members of the NRT1/PTR family (NPF), contributing to NO_3_^−^ and Cl^−^ translocation from the roots to the shoots through the xylem system, respectively. An apoplastic copper amine oxidase gene, *AO1* (ref. ^52^), which has been found to promote protoxylem differentiation, and a sulfated peptide gene, *CIF1* (ref. ^53^), involved in maintaining ion homoeostasis in xylem, were also included (Fig. 4d). In contrast, phloem development regulatory genes were enriched in light-induced modules; these genes included the *ALTERED PHLOEM DEVELOPMENT* (*APL*) gene, which plays a vital role in phloem–xylem patterning^54^. Both phloem cell proliferation and differentiation were impaired in *apl* mutants, and xylem development was inhibited by overexpression of *APL*^54^. We thus inferred that the perception of light signals in procambium cells regulated the transcriptional patterns of stem cells and affected the development strategy of phloem and xylem. Another six transcription factors whose expression patterns were the same as those of *APL*, *DOF5.6* and *MYR1* (ME21 and ME25) were also identified; these included *EFM*, *MYR2*, *PHL12*, *RL3*, *MJJ3.20* and *RL4* (Supplementary Fig. 8), which have possible important and novel functions in phloem development.

### Light promotes GC development by trajectory switching

Different from vascular cells, cotyledon epidermal cell subtypes underwent a tremendous status switch during de-etiolation from E.C1 to E.C2 (Fig. 1f). In contrast, the E.C3 cluster was relatively stable (Fig. 1f). Given the high expression of *FAMA*^55^ in E.C3 (Fig. 1d), we thus annotated the E.C3 cluster cells as GCs (Fig. 1b). The developmental trajectory of stomatal cell lineages has been illustrated clearly and verified via single-cell transcriptomic data^31,36,56^. The sequential expression of three stomatal lineage (SL)-specific basic helix-loop-helix (bHLH) transcription factors promotes the development of protoderm cells (PDC) into mature GCs. First, the expression of SPEECHLESS (SPCH) initiates the stomatal fate, helping to maintain the constant asymmetric division of meristemoid mother cells (MMCs) and the formation of meristemoid (M) cells^57^. The expression of the next main factor, MUTE, is a key transition event needed to acquire guard mother cell (GMC) identity from an M cell^56^. Subsequently, in this process, a GMC divides into two parts, ultimately completing the transition from young guard cells (YGCs) to GCs, where the third factor, FAMA^55^, is involved. Nonetheless, all trajectories were reconstructed for epidermal cells of light-grown seedlings.

**Fig. 5:**
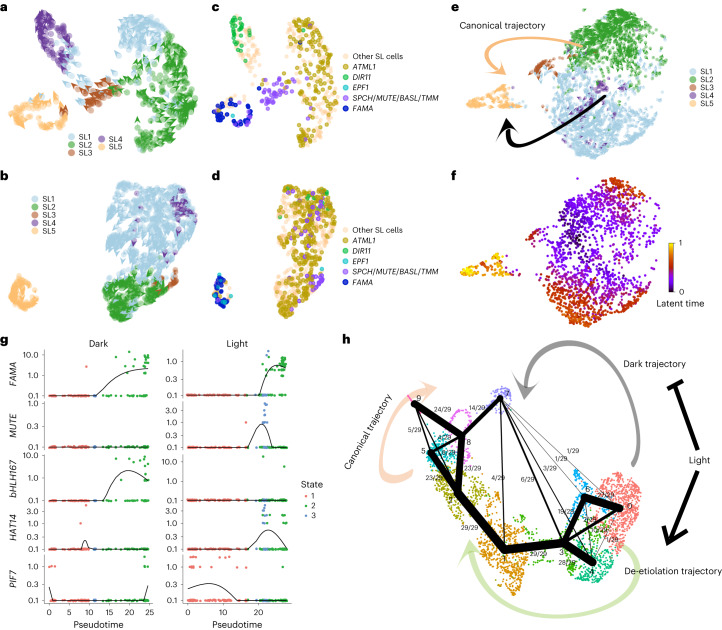
GC development networks under darkness and light. **a**, RNA velocity inferred for SL cells of light-grown samples. The colours indicate different subtypes. **b**, RNA velocity inferred for SL cells of dark-grown samples. The colours indicate different subtypes. **c**, Cell atlases for SL of Light samples. Transcript distributions of development factors in SL cells are highlighted by different colours. **d**, Cell atlases for SL of Dark samples. Two replicates of Dark samples are merged. Transcript distributions of development factors in SL cells are highlighted by different colours. **e**, Visualization of SL cells during the de-etiolation process. The orange and black arrows indicate canonical and dark-specific trajectories, respectively, for GC development. **f**, Latent time inferred for SL cells during the de-etiolation process; a darker colour represents earlier development stages. **g**, Expression trends of candidate regulators involved in SL development in dark and light conditions along the inferred pseudotime. **h**, Regulatory models for GC development. The black dots represent the respective cell clusters (0–9). Cells belonged to different clusters were coloured in respective colours: dark-grown precursors: 0, 4, 6; de-etiolating precursors: 1, 3; light-grown precursors: 2, 5; light-grown pavement cell: 9; light-grown GC: 8; dark-grown and de-etiolating GC: 7. The black edges between the black dots represent probabilities for cell state transitions. The size of the edges corresponds to the approval rate (the frequency of occurence for edges). The candidate genes involved in the respective trajectories are listed.

To resolve the development trajectory in darkness and changes induced by light, we first reconstructed the SL cell atlases in light and darkness (Fig. 5a,b). Epidermal cells collected from cotyledons of seedlings grown in constant darkness and constant-light conditions were combined to anchor corresponding cell types in Dark and Light (Methods). The SL cells were further clustered into five subtypes, and the development direction of each cell was inferred with spliced and unspliced transcripts (Fig. 5c,d and Methods). A series of genes involved in early SL development, such as *SPCH* and *BASL*, were transcribed specifically in SL3, indicating the M cell identity (Fig. 5c). SL4 and SL5 were annotated as pavement cells and GCs with the special distribution of *DIR11* (ref. ^36^) and *FAMA*^55^, respectively (Fig. 5c). SL1 and SL2 were classified as epidermal precursor cells because they border M cells in the opposite direction of mature cells (SL4 and SL5) on the UMAP atlas. Consistent with the trajectory inferred by cell identity (Fig. 5c), RNA velocity indicated that the development direction starts from SL2, proceeds through SL3 and finally develops into SL4 and SL5 (Fig. 5a), which is in concert with previous studies. Through the same pipeline, a different scene was depicted for SL cells under darkness (Fig. 5b,d). Pavement cells and M cells were distributed within epidermal precursor cell clusters. M cells (where *SPCH* was expressed) did not appear at the joint position of precursor cells and GCs, although M cells clustered with and in proximity to SL2. The trajectory direction inferred by RNA velocity indicated that GCs developed from precursors without passing the M cell states under darkness (Fig. 5b). Two distinct developmental trajectories towards GCs were illustrated more clearly with epidermal cells from de-etiolating samples. The development process begins with precursor cells with darker colour and proceeds to M cells (SL3) or results in the development of GCs (SL5) directly (Fig. 5e,f). Notably, cells on the direct path to GCs were enriched only in the samples of dark-grown seedlings (Supplementary Fig. 9). We thus inferred that epidermal cells could develop into GCs under darkness but via a completely different trajectory.

To explore how GC development proceeds in darkness and under light, cells in the canonical light trajectory (Supplementary Fig. 10a) and the dark-specific direct trajectory (Supplementary Fig. 10b) were separated from others and further ordered according to pseudotime. Candidate factors involved in respective development processes were identified and labelled on the merged epidermal cell atlas (Fig. 5g,h and Supplementary Fig. 10c). As a result, the atlas includes three paths, namely, dark-specific trajectory to GCs, canonical light trajectory to GCs and the de-etiolation trajectory of stomatal precursor cells (Fig. 5h). In darkness, transcription factors including *NAC3*, *ARF31* and *bHLH87* were differentially expressed along the trajectory to GCs (Fig. 5h). In addition, precursor cells underwent dramatic de-etiolation with the induction of *GL2*, *PDF2*, *ATML1* and *ICE* expression (Fig. 5h). The GCs of etiolated and de-etiolating seedlings (7) were similar to those of seedlings grown under constant light (8) but not exactly the same. Known GC-specific regulators such as *FAMA*, *DOF5.7* and *MYB60* were transcribed in both clusters; however, *bHLH167*, *bHLH92* and *DOF4.7* were specifically induced in the GCs of etiolated and de-etiolating seedlings (Fig. 5h). To validate the expression of *bHLH167*, *pbHLH167*:H2B-YFP reporter and *pFAMA*:CFP-FAMA^55^ were established^58^ and the signals were observed for dark- and light-grown seedlings (Methods). In darkness, the expression of *bHLH167* was restricted to GCs and no expression of *FAMA* was detected (Supplementary Fig. 11a). The signal of the *bHLH167* promoter was weaker in light-grown seedlings, while the expression of *FAMA* could be observed clearly in GMCs and GCs (Supplementary Fig. 11b). Crucial roles of candidate regulators in GC development and maturation under different light conditions need further studies.

### Cell-type-specific function of PIFs

**Fig. 6:**
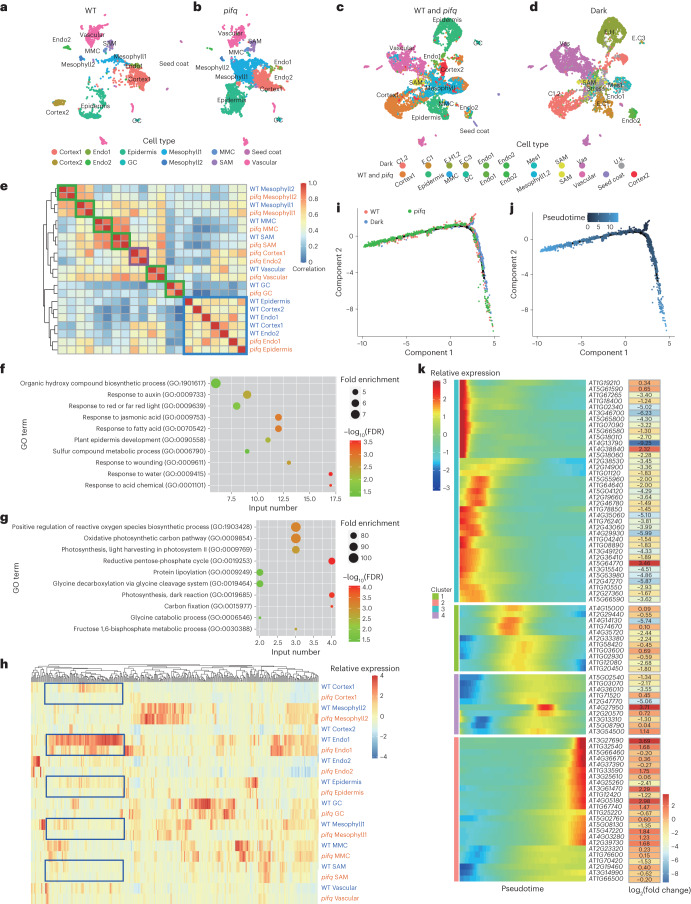
Cell-type-specific function of PIFs. **a**, Visualization of shoot cell types via UMAP for dark-grown WT. Cell types are colour coded. Annotations for each cell type are indicated. **b**, Visualization of shoot cell types via UMAP for dark-grown *pifq* mutant. Cell types are colour coded. Annotations for each cell type are indicated. **c**,**d**, Visualization of shoot cell types via UMAP by integration of WT and *pifq* atlas with Dark sample in the de-etiolating shoot atlas after batch-effect correction. Cell types are annotated for WT and *pifq*. The corresponding cell types are set in the same column. **e**, Correlation analyses for different cell types in WT and *pifq* with whole-genome expression patterns. The expression ratio (the number of cells where the gene is transcribed/the total number of cells) for each gene in each cell type was calculated and used for Pearson correlation analyses. Cell-type pairs highly correlated in two samples are indicated by green squares. Cell-type clusters without highly correlated pairs are indicated by purple and blue squares. **f**,**g**, GO enrichment results for down- (**f**) and upregulated (**g**) genes in cell type clusters surrounded by the blue square. **h**, The expression patterns of PIF direct target genes in different cell types of dark-grown WT and *pifq* shoot tissues. A cluster of targets with similar patterns in most cell types but highly expressed in endodermal cells are enclosed in blue rectangles. **i**,**j**, The development trajectory of epidermal cells for dark-grown WT and the *pifq* mutant. Corresponding samples (**i**) and pseudotime inferred by expression (**j**) are indicated by different colours. **k**, The expression of PIF direct target genes during guard cell development. PIF direct target genes with significant expression difference during guard cell development are identified. Relative expression levels of these genes for cells sorted by pseudotime are illustrated. The values of log_2_(fold change) (expression in the *pifq* mutant/expression in WT) calculated with bulk RNA-seq data are supplied on the right.

PIF transcription factors are core negative transcription factors involved in photomorphogenesis^59^. Quadruple *pif* mutants (*pifq*), lacking PIF-family members PIF1, PIF3, PIF4 and PIF5 (termed the PIF quartet), exhibited a constitutive photomorphogenic phenotype in darkness^60^. As factors transcribed in all cell types (Supplementary Fig. 12), the regulatory roles of PIFs in respective cell types are unclear. Another batch of protoplast isolation and scRNA-seq techniques were carried out for shoot tissue of dark-grown *pifq* and WT seedlings (Methods). After cell embedding and annotation were performed, major shoot cell types were identified and annotated on the atlas, mainly including vascular, epidermal, cortical and mesophyll cells (Fig. 6a,b). Compared with WT, the proportions of epidermal and mesophyll cells were increased, and vascular, endodermis and cortical cell proportions were reduced (Supplementary Fig. 13). Intriguingly, a subtype of cortical cells (Cortex2) was observed solely in WT (Fig. 6a,b and Supplementary Fig. 13). Integrating these data with Dark samples in the de-etiolating atlas (Methods) revealed that Cortex2 cells were similar to perturbed cells (U.k., Fig. 6c,d). Whole-genome expression patterns for each cell type of *pifq* and WT were calculated and used for correlation analyses (Methods). Similar cell-type pairs were observed for the same identities between two samples, including mesophyll, vascular, MMC, SAM and GC (Fig. 6e), indicating similar states with and without PIFs. In contrast, a group of cell types, comprising epidermis, cortex and endodermis, showed higher correlation with different cell types in the same sample, but lower correlation with the same cell type between samples (Fig. 6e). Differential expression genes were identified between *pifq* and WT for this group of cell types (Methods). With the mutation of PIFs, while expression inhibition of genes was enriched in the auxin-responsive pathway (Fig. 6f), the increased expression was observed broadly in photosynthesis genes (Fig. 6g).

Light signalling works in concert with phytohormone signalling cascades to regulate plant development^61^. Why were auxin-responsive genes downregulated in specific cell types? Spatiotemporal expression patterns of genes involved in phytohormone biosynthesis and signalling during the de-etiolation process are illustrated in Supplementary Figs. 14 and 15. In shoot cells, the distribution of cell-type-specific genes involved in abscisic acid (ABA) and auxin biosynthesis exhibited a preference for vascular parenchymal cells (Vas3), and spatiotemporally restricted genes involved in jasmonic acid, gibberellic acid and ABA signalling were enriched in procambial cells (Vas1) (Supplementary Fig. 14 and Supplementary Table 10). Notably, a number of auxin signalling genes were specifically expressed in etiolated hypocotyl epidermal and cortical cells (E.H1 and C1). Intriguingly, there were 19 *SMALL AUXIN UP RNA* (*SAUR*) genes identified as E.H1- and C1-specific genes, encoding a group of sequence-related genes whose expression was rapidly induced by auxin^62,63^ (Supplementary Fig. 16).

To investigate the direct function of PIFs in cell state changes, expressions of direct target genes of PIFs are illustrated in cell-type resolution in Fig. 6h. More than one-fourth (71/276) of high-expression PIF direct target genes were highly transcribed in endodermal cells, although the mutation of PIF-induced expression decrease was similar among all cell types (Fig. 6h). Auxin-responsive genes, including *SAUR14*, *SAUR19*, *SAUR23*, *SAUR25*, IAA3, *IAA19*, *GH3.1* and *PRE5*, belong to this gene set (Supplementary Table 11). In addition, significant expression differences were observed specifically in MMCs and GCs (Fig. 6h); hence we analysed the development trajectory of epidermal cells for *pifq* and WT (Fig. 6i,j). Stomatal lineage cells from *pifq* samples were enriched in mature states of GC development (Fig. 6j and Supplementary Fig. 17). Differential expression genes in GC development were identified and the expression patterns of PIF direct target genes are illustrated in Fig. 6k. These targets were transcribed in specific stages along GC development and maturation. Moreover, the expression induction was enriched for targets highly transcribed in mature stages in *pifq* mutants (Fig. 6k). In darkness, PIFs promoted auxin-responsive genes in endosperm but inhibited GC maturation factors in stomatal lineage cells, accomplishing independent regulation by cell-type-specific expression of targets.

### Disparate responses to light in distinct shoot cell types

Different cell types have varying degrees of plasticity, especially in shoot tissues (Supplementary Figs. 18–25). Cell states and trajectories of the vascular system are quite stable during de-etiolation (Supplementary Fig. 22). However, dramatic transcriptomic reprogramming was observed in mesophyll cells, hypocotyl, epidermal and cortical cells from dark status to light status (Supplementary Figs. 18–20). Cotyledon epidermal cells also underwent strong changes, while GCs were relatively stable (Supplementary Fig. 21). The switch in cell status could be resolved into DEGs among light exposure time in each cell type. On the other hand, a total of 7,857 DEGs were identified for each cell type among light exposure timepoints (Supplementary Table 12). In most cell types except mesophyll cells, the largest DEG set was identified after 24 h of light in the de-etiolation seedlings (Supplementary Fig. 26a). Most genes were differentially expressed in the mesophyll cells of dark-grown and early-stage de-etiolation seedlings compared with the other timepoints (Supplementary Fig. 26a), indicating that mesophyll cells constitute the early and active phase of light perception and signalling. More than 45% (3,589) of the DEGs were cell-type specific and only 614 genes were identified as DEGs in all seven shoot cell types (Supplementary Fig. 26b), which were enriched in photosynthesis genes (Supplementary Table 13).

Light perception and signalling in different cell types induced the expression of extensively different DEGs and caused various subsequent developmental switches. Light signals affected cell fate decisions by regulating cell type-specific core factors. The cell developmental trajectories constructed here and the candidate factors involved will facilitate the identification of novel cell fate regulators and lead to light signalling studies at the single-cell resolution.

## Discussion

Light-induced cell state transitions and developments, as well as related transcriptional regulations, are the spotlights of this study. Previous shoot cell atlases^35,37^ have made great progress in cell-type identification and cell trajectories construction, similar to a high-resolution picture in one moment. However, it is unresolved whether cells from dark-grown seedlings share the same developmental trajectories as light-grown ones. In this study, we focused on the de-etiolation process, constructing cell atlases in a continuous mode. With the combination of time-series atlases, we restored the developmental process in respective cell types. We observed the trajectory and regulatory factors for guard cell development, vasculature and cortical cells in dark and de-etiolating processes. A good illustration of genome-wide expression patterns in different cell types after light exposure facilitated our understanding of the common machinery affecting respective cell development processes.

As our data suggested, the response patterns were dramatically distinct in the different cell types during de-etiolation. For the two cell populations with intrinsic trajectories, epidermis and vasculature, we have updated the overview of cell development. The differentiating atlases have been constructed for both light and dark-grown seedlings (Fig. 4a–d). The number of M cells is too small or the states were different between dark and light-grown samples; hence the transition state from precursors to GCs was not observed. In the present study, we propose that the production of GCs in darkness is regulated by a new set of regulators. Therefore, it is necessary to resolve the function of regulators in darkness and their responses to light signals. The expression of positive regulators in xylem development was inhibited within a short time of light radiation, and positive regulators in phloem development were induced gradually (Fig. 4c), although it was reported that light-grown hypocotyls possess more mature reticulate and pitted metaxylem compared with dark-grown tissues^25^. The phloem cell system needs to develop more vigorously to promote photosynthate transport from apical parts after light exposure when photosynthesis starts. The fine-tuning of the development strategy by light signals makes it possible to regulate plant architecture immediately.

Chloroplast biogenesis and development are the most critical steps during de-etiolation. The complicated processes are regulated by several thousand nuclear genes and ~100 chloroplast genes^64^ (Supplementary Table 14). We analysed two sets of chloroplast biogenesis genes: gene set (1) comprising causal genes for abnormal phenotypes in chloroplast biogenesis and development in *Arabidopsis*^65^ (Supplementary Fig. 27) and gene set (2) comprising *Arabidopsis* orthologues of maize genes mapped with a photosynthetic mutant library^64^ (Supplementary Fig. 28). Most genes were induced in all seven shoot cell types and had higher expression in mesophyll cells (Supplementary Figs. 27 and 28). However, some important regulatory genes showed different expression patterns with the process of photosynthesis (Supplementary Fig. 27). For example, one of the photomorphogenesis regulator B-box zinc finger protein 22 (AT1G78600) was transcribed most strongly in E.H but not in Mes cells. The expression of some factors, including phytochrome A (phyA, AT1G09570), was inhibited by light. Intriguingly, genes co-expressed with phyA were enriched in SAM, indicating the important roles of cell differentiation in regulation. To extensively explore light responses in chloroplast, 1,720 chloroplast core nuclear proteins^66^ (Supplementary Fig. 29) were analysed (Supplementary Fig. 29). Similar to biogenesis regulators, most chloroplast genes were induced globally, but with different expression preferences. Genes repressed by light were also identified, which were likely to regulate photosynthesis in a more complicated way similar to phyA. Compared with the background, globally light-induced genes (ME1) were significantly enriched in chloroplast-related genes (Supplementary Fig. 30), which is in accordance with the photosynthesis process.

Apart from the above findings, we have identified cell-type-specific responsive genes to light (Fig. 3 and Supplementary Tables 6–8). However, why did the different cell types respond differently? To explain the cell-type response heterogeneity, we focused on *HY5* and *PIF* genes, two families of core transcription factors that function oppositely in photomorphogenesis^15^. The expression pattern of *HY5* was universally induced in response to light and peaked after 1 h of light (Supplementary Fig. 31). *PIF* genes were mainly transcribed in the aerial portion of *Arabidopsis* seedlings and no obvious preferential expression was detected for *PIF1*, *PIF3*, *PIF4* and *PIF5* within de-etiolating shoot cell types (Supplementary Fig. 12). However, the direct target genes of HY5 (ref. ^20^) and PIFs^45^ exhibited diverse expression patterns during de-etiolation (Supplementary Fig. 32). Nearly 70% (204/297 for HY5 and 233/338 for PIFs) of the target genes were identified as exhibiting spatiotemporally specific expression (Supplementary Tables 5 and 6). Furthermore, mutation of PIFs influenced transcriptional states at different levels in different cell types (Fig. 6e). We thus propose that epigenetic modification and chromatin structure differ among the cell types, or that there are cell-type-specific co-factors involved. Thus, the application of multiple omics for different cell types will help us to precisely resolve and predict cell responses to external signals.

In summary, we generated a time-series gene expression map of de-etiolated seedlings and their response to light. We finely dissected light-responsive genes, cell trajectories and core regulators of light signal transduction for the different cell types, leading to an improved understanding of the relationship between skotomorphogenesis and photomorphogenesis. Comparing atlases of WT and the *pifq* mutant, we uncover cell-type-specific effects of PIFs and their independent regulatory mechanisms. These transcriptional changes in response to light in cell-type resolution could facilitate studies to fine-tune mechanisms of light signalling.

## Methods

### Culture and sampling of *Arabidopsis* seedlings

*Arabidopsis* ecotype Columbia (Col-0) seeds were surface sterilized and subjected to 4 °C for 2 d in complete darkness, after which they were grown on 0.3% sucrose Murashige and Skoog media (Sigma-Aldrich, M5519) supplemented with 7% Phytogel (w/v; Sigma-Aldrich, P8169) and 0.05% MES hydrate (w/v; Emresco, E196) (pH 5.7) on vertically oriented plates. The seeds were exposed to constant white light for 4 h at 21 °C to synchronize germination and then placed under darkness or white light for 5 d and sampled to constitute constant-dark (Dark) and constant-light (Light) materials. The dark samples were then transferred to white light for 24 h to promote de-etiolation. Etiolated seedlings illuminated for 1 h, 6 h and 24 h were sampled, constituting D2L1h, D2L6h and D2L24h materials. Two replicates of Dark samples for shoot and root were sequenced. Therefore, a total of 12 samples were used for construction of the de-etiolating atlas. Dark-grown shoot tissues were also sampled to obtain single-nucleus (sn) data. For construction of the pifq atlas, a batch of *pifq* seedlings and wild-type (Col-0) seedlings were cultivated in the same manner as described above.

### Library construction for scRNA-seq

For cell wall digestion, root and shoot tissues were collected and placed into cell culture plates that contained enzyme solution: 0.4 M mannitol (GPC, AC065), 0.02 M MES (Emresco, E196), 0.02 M KCl (Sigma-Aldrich, V900068), 0.01 M CaCl_2_ (Sigma-Aldrich, V900266), 0.1% (w/v) BSA (Sigma-Aldrich, B2064), 1.5% (w/v) cellulase (Onozuka, R-10) and 0.4% (w/v) macerozyme (Onozuka, R-10). The plates were rotated at 80–100 r.p.m. at room temperature. Light-grown shoot tissues were incubated for 1 h and other samples were incubated for 2 h. The protoplast solution was then strained through a 70-μm filter (Falcon, 352350), followed by a 40-μm filter (Falcon, 352340). The filtered solution was centrifuged at 100 × *g* for 7 min, after which the pelleted protoplasts were resuspended in 5 ml of washing solution (enzyme solution without enzyme or CaCl_2_) and then centrifuged at 100 × *g* for 4 min. The pelleted protoplasts were resuspended in 300–1,000 μl of washing solution until the desired cell concentration was reached. The manipulation for dark-grown samples was carried out in a dark room and others were in normal light conditions.

For single-nucleus data, the shoot tissues of dark-grown seedlings were frozen in liquid nitrogen, added to nuclear extraction buffer, fragmented with a gentleMACS Octo dissociator (Miltenyi Biotec, 130-095-937), and finally filtered and suspended in 300 μl of 10x wash buffer for nuclear isolation^67^. The protoplast or nuclear suspension was loaded into Chromium microfluidic chips with v.3.1 chemistry and barcoded with a Chromium Controller (10x Genomics). RNA from the barcoded cells was subsequently reverse-transcribed, and sequencing libraries were constructed with reagents from a Chromium Single Cell v.3.1 reagent kit (10x Genomics) according to manufacturer instructions. Sequencing was performed on an Illumina HiSeq 4000 according to manufacturer instructions.

### Calculation of correlation coefficients

For the single-cell data, each expression matrix was normalized with the NormalizeData function of the Seurat package with normalization.method = ‘RC’. The average expression of each gene in all the cells was calculated with the rowMeans command. For the bulk data, expression matrices were downloaded from the GEO database. The expression data were merged for both the single-cell data and bulk data for the overlapping genes. The expression matrix was used to calculate a Pearson correlation coefficient matrix and visualized using pheatmap.

### Construction of cell atlases of de-etiolated seedlings

The raw scRNA-seq dataset was first analysed using Cell Ranger 3.0.2 (10x Genomics). The genome (TAIR 10) and GTF annotation (Araport11) files of *Arabidopsis* were downloaded from https://www.arabidopsis.org/. Reference and annotation indices were obtained using the cellranger mkref command. The reads were aligned to the reference sequence, and expression levels were determined for each cell and gene using the cellranger count command. The gene–cell matrices were subsequently loaded into the Seurat^68^ package (v.3.2.0), which was implemented in R (v.4.0.2). To remove dead cells and dissociative RNAs, we filtered cells with unique gene counts of <300. To remove doublet cells, cells with unique gene counts >5,000 for root samples and >4,000 for cotyledon samples were also filtered and removed. Cells with >5% mitochondrial sequences, >3% chloroplast sequences for root samples and >20% chloroplast sequences for cotyledon samples were filtered and removed. In addition, only genes expressed in at least three individual cells were retained. The clean and normalized data obtained above were combined with the merge command, scaled data were further calculated for the detection of highly variable genes using the ‘mean.var.plot’ method of the FindVariableFeatures command, and genes were filtered in accordance with dispersion.cutoff=c(1, Inf) and with mean.cutoff=c(0.01,3). The expression levels of the feature genes selected above were subjected to principal component analysis (PCA) dimension reduction. Then, cells from different batches were embedded with UMAP and *t*-distributed stochastic neighbor embedding (TSNE) in the same two-dimensional space. The resulting cell clusters were classified using FindNeighbors and FindClusters.

### Construction of cell atlases for snRNA data and *pifq* data

Raw sequencing reads mapping, quality control and dimensionality reduction were manipulated following the pipeline described above. The expressions of marker genes used for annotation of the de-etiolating atlas were illustrated on the atlases and corresponding cell identities were annotated. The *pifq* atlas was directly merged with that of the wild type from the same batch as little batch effect was observed. Cell expression ratios for each gene were calculated for cell types in wild type and the *pifq* mutant using manual R (v.4.02) scripts. Correlation coefficients were obtained using the ‘cor’ function in R. The combination of snRNA-seq data and scRNA-seq data for dark-grown shoot samples and combination of *pifq*, WT and Dark samples were carried out using feature anchoring and the integrateData function in Seurat^68^.

### Identification of light-responsive genes

The Dark, D2L1h, D2L6h, D2L24h and Light *Arabidopsis* seedlings were cut into cotyledon, hypocotyl and root parts, after which total mRNA was extracted from each sample with RNeasy plant mini kit (50, Qiagen, 74904) and prepared for RNA-seq library construction. We downloaded available cotyledon and hypocotyl RNA read sequences^20^ from the NCBI GEO database. The raw reads were subjected to quality control with Trimmomatic (v.0.39)^69^, the clean reads were aligned to the TAIR 10 reference genome using HISAT2 (v.2.2.0)^70^ and the unique mapped reads were selected for expression-level quantification via SAMTOOLS (v.1.6)^71^ and StringTie (v.2.2.1)^72^. The transcripts per million (TPM) value for each sample was merged using a Perl (v.5.32) script. We carried out a *t*-test for each sample of de-etiolated and light-grown seedlings, with the corresponding sample of dark-grown seedlings used as a control. Genes whose expression exhibited a fold change of >2 and a *q*-value of <0.05 for at least one comparison were identified as light-responsive genes. For the identification of cell-type-specific DEGs for scRNA-seq data, we first extracted Seurat objects of the seven cell types. DEGs were calculated using FindMarkers for each time exposure by setting pt.1. Genes with pt.1 > pt.2 were considered upregulated, while those with pt.1 < pt.2 were considered downregulated.

### Identification of spatiotemporal markers

Shoot and root cells were reanalysed independently with the Seurat pipeline mentioned above. First, we identified cell-cluster-enriched genes using the FindAllMarkers function with the ‘logfc.threshold=0.1, only.pos=TRUE, min.pct=0.1’ parameters. The significant cluster-specific genes were further screened using a pt.2 < 0.1 and an *e*-value < 1 × 10^−30^. Marker genes shared by more than one cluster were further screened using an *e*-value < 1 × 10^−100^. For identification of spatiotemporal expression modules for de-etiolated shoot tissues, we annotated cell clusters manually into seven cell types: Mes, SAM, En, E.C, E.H, C and Vas. The mean expression of each gene for the seven cell types and six samples (D2L1h, D2L6h, D2L24h, Light and two replicates each of the Dark materials) was calculated using normalized UMI counts. The resulting expression matrix was analysed using the WGCNA (v.1.69)^44^ pipeline with the default filtration process.

### Construction of stomatal cell lineage trajectories

The Seurat objects of E.C and E.H cells from the Light and Dark samples were extracted and used for reconstruction of cell atlases, and hypocotyl cell clusters were deleted according to the expression of marker genes (Supplementary Fig. 6). Stoma lineage cell data for each sample were normalized and used for variable features identification. Feature genes as anchors were identified with the FindIntegrationAnchors command and used for correction of light-induced batch effects employing IntegrateData. The resulting data were classified into five clusters (SL1–5). Then, the cells of the light- and dark-grown seedlings were separated and used to reconstruct the cell trajectory with their respective feature genes with Seurat functions. RNA velocity and latent time were estimated using ScVelo (v.0.2.3)^73^. Stomatal cell data of de-etiolated seedlings were extracted following the same methods as described above; the Seurat-clustered expression matrix was imported into monocle2 (v.2.14.0)^74^, and cluster-specific genes were subsequently identified using the differentialGeneTest function. These genes were used as features to reconstruct cell trajectories with Seurat. The developmental trajectories between cell clusters were modelled using Slingshot (v.1.8.0)^75^ 29 times with the first 2 to 30 principal components. Candidate core regulators involved in stomatal cell lineage development were identified with monocle2 via the ‘fullModelFormulaStr = ~sm.ns(Pseudotime)’ parameter.

### Validation of spatiotemporal markers

To investigate the expression patterns of marker genes in specific cell types at specific development stages, we established a tissue-specific expression system. The seed-specific *AT2S3* promoter was used to drive the expression of DsRed2 (ref. ^76^). Promoters of spatiotemporal marker genes were used to drive the expression of GFP^77^. Hypocotyl sections were prepared by embedding seedlings in 2% low-melting agarose (V2111, Promega). Transverse sections were generated with a blade after coagulation. For *bHLH167*, the upstream promoter sequences were amplified via PCR with KOD FX DNA polymerase (Toyobo, KFX-101) and cloned in front of the H2B-YFP coding sequence. The resulting *pFAMA*:CFP-*FAMA* construct was then inserted into the same vector to label the location of *FAMA*.

### Reporting summary

Further information on research design is available in the Nature Portfolio Reporting Summary linked to this article.

### Supplementary information

Supplementary Information Supplementary Methods and Figs. 1–37.

Reporting Summary

Supplementary Tables Supplementary Tables 1–15.

## Data Availability

The fastq files for single-cell and bulk RNA-seq are available. The data can be obtained from the National Genomics Data Center (PRJCA016521). The spatiotemporal expression patterns in the de-etiolating atlases are available at http://182.92.183.62:4576/ or http://www.pku-iaas.edu.cn/list_38/64.html.

## Code availability

All custom codes used in this study are available at http://182.92.183.62:4576/.
