# Supplementary material for: Time series single-cell transcriptional atlases reveal cell fate differentiation driven by light in Arabidopsis seedlings
Source: Nat Plants. 2023 Oct 30;9(12):2095–109. doi: 10.1038/s41477-023-01544-4 (PMC10724060; doi:10.1038/s41477-023-01544-4)
Supplement: Supplementary file 1 — Supplementary Methods and Figs. 1–37. [file 41477_2023_1544_MOESM1_ESM.pdf]

nature plants

Resource https://doi.org/10.1038/s41477-023-01544-4

Time series single-cell transcriptional atlases reveal cell fate differentiation driven by light in *Arabidopsis* seedlings

In the format provided by the authors and unedited

Table-of-Contents:

Supplementary Methods: 4

Construction of Cell Atlas with Shoot and Root Cells Merged Directly 4

Assessment of Sample Representativeness. 4

Cell Type Annotation and Validation 5

Reference: 8

Supplementary Figures: 10

Figure S1. Correlation Coefficient of RNA-Seq Data. 10

Figure S2. The Principal Component Analysis of ScRNA-Seq during De-Etiolation. 11

Figure S3. The Principal Component Analysis of Bulk RNA-Seq for Three Organs during De-Etiolation. 11

Figure S4. Visualization of Seedling Cells Using UMAP. 12

Figure S5. Expression Patterns of Cell Markers on the De-Etiolating Atlases. 13

Figure S6. Expression Patterns of Hypocotyl Markers in Shoot Atlas. . . . 14

Figure S7. Proportion of Dividing Cells in De-Etiolation Process. 15

Figure S8. Expression Patterns of Candidate Vasculature Development Regulators in Vascular Cells. 15

Figure S10. Trajectories of GCs. 16

Figure S11. Expression of *bHLH167* in Epidermis for Seedlings Grown in Constant Dark for 3 Days. 17

Figure S12. Expression Patterns of PIFs. 18

Figure S13 Cell Proportions for Dark-Grown WT and *pifq* Shoot. 19

Figure S14 Distribution of Spatiotemporally Specific Genes Involved in Hormone Biosynthesis (Upper) and Signaling (Lower) in Root. 19

Figure S15 Distribution of Spatiotemporally Specific Genes Involved in Hormone Biosynthesis (Upper) and Signaling (Lower) in Shoot. 20

Figure S16. Spatiotemporal Expression Patterns of *SAUR* genes during De-Etiolation. 20

Figure S17. The Development Trajectory of Epidermal Cells for Dark-Grown WT and the *pifq* Mutant. 21

Figure S18. The Development Trajectories of Mesophyll Cells during De-Etiolation. 22
Figure S19. The Development Trajectories of Hypocotyl Epidermal Cells during De-Etiolation. 22
Figure S20. The Development Trajectories of Cortex Cells during De-Etiolation. 23
Figure S21. The Development Trajectories of Cotyledon Epidermal Cells during De-Etiolation. 23
Figure S22. The Development Trajectories of Vascular Cells during De-Etiolation. 24
Figure S23. The Development Trajectories of Endodermis Cells during De-Etiolation. 24
Figure S24. The development trajectories of SAM Cells during De-Etiolation. 25
Figure S25. The Velocities for Seven Cell Types during De-Etiolation. . . 26
Figure S27 The expression patterns of gene set 1 involved in plastid biogenesis.. 28
Figure S28 The expression patterns of gene set 2 involved in plastid biogenesis.. 29
Figure S29 The Expression Patterns of Genes Involved in Plastid Biogenesis. 30
Figure S30 Radar plots showing the distribution of co-expression modules of chloroplast biogenesis genes. 30
Figure S31. Relative Expression of *HY5* during De-Etiolation in Each Shoot Cell Type. 31
Figure S32. Relative expression for direct targe genes of HY5 and PIFs during de-etiolation. 31
Figure S33. Expression Patterns of Shoot Cell Markers in Single Nucleus Atlas.. 32
Figure S34. Cell Cluster Correspondence before and after Batch Effect Correction. 33
Figure S35. Annotation of Rcluster 24. 34

Figure S36. A diagram Indicating the Location of the Seedling for Respective Cell Types. 35
Figure S37 Expression Patterns of Cell Markers on the *pifq* atlas. 36

Supplementary Methods:

Construction of Cell Atlas with Shoot and Root Cells Merged Directly

The batch effect is one of the most obstinate issues for scRNA-seq data and is induced by technical and random variation during single-cell isolation and low-coverage sequencing[1]. However, correction of batch effects can also lead to overfitting and loss of true biological variation. In this study, the highly reproducible single-cell transcriptomic data we obtained could be merged directly (Methods). The merged data was depicted with dimension reduction of highly variable genes, and all cells from root and shoot tissues were classified into 27 metaclusters (Figure S4a). Root and shoot samples were clearly separated into a left part and a right part of the atlas (Figure S4b). Therefore, discrepancies among organs play a main role in transcriptional differences among cells. Consistent with the similarity among samples revealed by the sum of the UMI counts of all cells (Figure S2), the cell constituents of *Arabidopsis* roots differed slightly among different the light conditions, even between constant darkness and constant light (Figure 1f). The root growth inhibition during de-etiolation might be induced by direct light radiation[2]. In contrast, the shoot atlases of dark-grown seedlings were sharply different from those of light-grown ones, and the cell states were gradually changed during the de-etiolation process (Figure 1c and Figure S2).

Assessment of Sample Representativeness

To validate the representation of the single-cell data, the UMI counts for each gene were summed and normalized for the respective samples to calculate correlation coefficients (Methods). Comparing them with published single-cell transcriptomes[3-5], as well as tissue-specific bulk transcriptomes for *Arabidopsis* seedlings[3,6], we found that the shoot and root samples clustered separately (Figure S1). Root single-cell samples strongly correlated with each other, not only among the data in this study but also within previously published ones (Figure S1), indicating that the transcriptional status of root samples is highly stable. In contrast, the shoot UMI data clustered into two clades. The shoot samples from the seedlings grown under light and the those exposed to light for 24 hours were significantly correlated with the bulk transcriptomes of the cotyledons of the seedlings grown under light, while the samples of the dark-grown seedlings and initial stages of samples of the de-etiolating seedlings clustered together (Figure S1). The different patterns of the shoots and roots were in accordance with the bulk transcriptomic data (Figure S1 and Figure S2) and de-etiolating seedling phenotypes (Figure 1). The cotyledons of the dark-grown seedlings changed progressively under light and ultimately developed extraordinarily similarly to the cotyledons of the light-grown seedlings after 24 hours. On the other hand, there was no dramatic difference among root tissues during de-etiolation (Figure 1c and 1f). The distinct status of shoot single-cell transcriptomes compared to tissue-specific bulk data might be induced by the combination of cotyledon and hypocotyl cells (Figure 1,

Figures S1 and S2). In summary, the scRNA-seq data in this study were found to be sufficiently representative and repeatable.

Cell Type Annotation and Validation

In this study, published shoot tissue atlas[7-9] as well as tissue-specific transcriptomic data[10] facilitated annotation of most shoot cell clusters. According to known spatial markers (Table S4 and Figure S5b), the identities of seven vasculature subtypes have been verified properly. Specific expression of *VASCULAR-RELATED NAC-DOMAIN 2* (*VND2*)[11], *SWEET11*[7], *ATPase 3* (*AHA3*)[12] and *SIEVE ELEMENT OCCLUSION B* (*SEOB*)[13] were used for annotation of xylem (Vas2), vasculature parenchymal cells (Vas3), phloem companion cells (Vas5) and sieve elements (Vas7) (Figure 1a). Procambium/provascular cells (Vas1), dividing provascular cells in G2-M phase (Vas4) and S phase (Vas6) were identified by *OCTOPUS* (*OPS*)[14] and cell cycle markers[9]. The identities of mesophyll (Mes), epidermis (E), endodermis (En) and shoot apical meristem (SAM) cell clusters have been verified by specific expression of *EPIDERMAL PATTERNING FACTOR 9* (*EPFL9*)[15], *MERISTEM LAYER 1* (*ATML1*)[16], *ACTIN DEPOLYMERIZING FACTOR 9* (*ADF9*)[9] and *SHOOT MERISTEMLESS* (*STM*)[17], respectively (Figure 1b and Figure S5). Epidermal cells were further classified as cotyledon epidermis (E.C) and hypocotyl epidermis (E.H) by hypocotyl markers, including *heavy metal-associated isoprenylated plant protein 31* (*HIPP31*)[10] (Figure S6). Expression enrichment of hypocotyl highly expressed genes have been detected not only in E.H and vasculature cells, but also in E.H-adjacent clusters (scluster 4, 19) and a E.H distant cluster (scluster 19) (Figure S6). We further constructed reporter lines of high expression genes for possible E.H and the unannotated hypocotyl clusters (scluster 4, and 14), because of lacking hypocotyl cell type markers. One of the epidermis-highly expressed gene *AT4G18970* were selected for E.H validation, whose specific promoter activity was confirmed in the epidermal cells in the intersect section of hypocotyls (Figure 2a). The promoter activities of highly expressed genes for scluster 4 (*AT3G05150*) and 11 (*AT1G78450*) were captured at cortical cells in hypocotyls of dark- (Figure 2b) and light-grown seedlings (Figure 2c). Although scluster 4 and 11 were distant clusters in the shoot atlas, they were both annotated as cortex cells (C) (Figure 2b and 2c), but in disparate states.

No valid spatial marker gene was identified for scluster 3, and high expression of heat shock genes was detected in those cells (Table S5); thus, we classified them as perturbed cells (U.k.) produced during enzyme digestion processes (Methods). The long-term protoplast isolation process is often doubted and discussed for its influence on cell status. To validate the accuracy of the atlases constructed with the protoplast data, an extra single-nucleus RNA-seq library for shoot tissue of dark-grown seedlings was sequenced (Methods). The single nucleus atlas was generated from raw reads after quality control and dimensionality reduction (Methods). The expression patterns of spatiotemporally restricted genes identified with the protoplast data were illustrated on the single nucleus atlas for annotation and comparison (Figure S33a and Figure S33b). Significant differences were observed comparing to shoot atlas in dark conditions

directly, because of large batch effect from two different libraries. However, the cell types were matched perfectly between two methods after correction of batch effect (Figure S33c). Therefore, the protoplast isolation process had little influence on clustering and annotation for cell types. At the same time, medians of 1,060 transcripts for shoot cells and 1,709 for root cells could be sequenced from single protoplasts, providing more than twofold more informative UMI counts than could single nuclei (with a median of 628). We thus confirmed that the single-protoplast transcriptomic data were reliable and information rich.

In general, shoot tissues undergo tremendous changes in both phenotype and expression status during de-etiolation; thus, several sclusters have been identified as the same cell type. To validate that these distributed sclusters were in fact different states of one cell type, we applied integration processes to regress the light-induced batch effect[18]. As expected, some sclusters were merged together, especially for the Mes, E and cortex (Figure S34), indicating that these cell types were particularly sensitive to light.

Root cell type annotation was facilitated by the use of previous root atlases. The expression patterns of one root tip cell atlas[19] were most similar to our cell type expression patterns. Mapping the top 20 enriched expressed genes in each cluster to the expression data resulted in annotations for the majority of rclusters (Figure S5a, and Table S4). Intriguingly, lateral root cap cells in this study were assigned to multiple distributed rclusters. To validate the identity, we constructed promoter reporter lines for the lateral root cap subtype-specific genes *AT1G53708* and *AT4G13890.* Although the expression region varied in the atlases of dark- and light-grown seedlings, both promoters were actively restricted to the root cap (Figure 2d and 2e), revealing that the annotations were accurate. Except for the validated root cell types described above, the identities of some rclusters were unresolved. A batch of shoot high expression genes were strongly transcribed in rcluster 24 (Figure S35a). A search of these cells within the combined atlas indicated that many cells of rcluster 24 were allocated to the area of shoot tissues (Figure S35b). It is possible that these shoot cells were retained at the sampling steps. The pile of cells at the center of the root map was profiled for marker gene identification, but no differential expression genes were screened out. However, multiple spatially restricted transcripts for different cell types were detected simultaneously in this area (Figure S5a), indicating that doublets might be enriched in these clusters. Finally, cells of rcluster 17 were enriched with stress-related transcripts (Table S6), which possibly occurred in damaged cells, such as those of shoot cluster 3, produced during cell wall degradation. Collectively, the finely annotated seedling de-etiolating atlas has been accomplished (Figure S36).

The annotation for the shoot atlas constructed for dark-grown seedlings of wild type and the *pifq* mutant was mainly according to makers used in annotation of shoot cell clusters (Figure 1d and Figure S37). The correspondence of cell clusters between the *pifq* atlas and the Dark atlas was also informative for cell type annotation (Figure 6c and Figure 6d). The cluster annotated as Cortex2 by cortical markers (Figure 6a and

Figure S37) was likely a cluster of perturbed cortical cells as the cell state was similar to the U.k. cluster.

Reference:

1. Haghverdi, L., Lun, A.T.L., Morgan, M.D., and Marioni, J.C. Batch effects in single-cell RNA-sequencing data are corrected by matching mutual nearest neighbors. *Nat Biotechnol.* 36, 421–427 (2018).
2. Silva-Navas, J., Moreno-Risueno, M.A., Manzano, C., Pallero-Baena, M., Navarro-Neila, S., Téllez-Robledo, B., Garcia-Mina, J.M., Baigorri, R., Gallego, F.J., del Pozo, J.C. D-Root: a system for cultivating plants with the roots in darkness or under different light conditions. *Plant J.* 84(1):244-55 (2015).
3. Denyer, T., Ma, X., Klesen, S., Scacchi, E., Nieselt, K., and Timmermans, M.C.P. Spatiotemporal Developmental Trajectories in the *Arabidopsis* Root Revealed Using High-Throughput Single-Cell RNA Sequencing. *Dev Cell.* 48, 840-852.e5 (2019).
4. Zhang, T.Q., Xu, Z.G., Shang, G.D., and Wang, J.W. A Single-Cell RNA Sequencing Profiles the Developmental Landscape of *Arabidopsis* Root. *Mol Plant.* 12, 648–660 (2019).
5. Liu, Z., Zhou, Y., Guo, J., Li, J., Tian, Z., Zhu, Z., Wang, J., Wu, R., Zhang, B., Hu, Y., et al. Global Dynamic Molecular Profiling of Stomatal Lineage Cell Development by Single-Cell RNA Sequencing. *Mol Plant.* 13, 1178–1193 (2020).
6. Burko, Y., Seluzicki, A., Zander, M., Pedmale, U.V., Ecker, J.R., and Chory, J. Chimeric Activators and Repressors Define HY5 Activity and Reveal a Light-Regulated Feedback Mechanism. *Plant Cell* 32, 967–983 (2020).
7. Kim, J.Y., Symeonidi, E., Pang, T.Y., Denyer, T., Weidauer, D., Bezrutczyk, M., Miras, M., Zöllner, N., Hartwig, T., Wudick, M.M., et al. Distinct identities of leaf phloem cells revealed by single cell transcriptomics. *Plant Cell* 33, 511–530 (2021).
8. Lopez-Anido, C.B., Vatén, A., Smoot, N.K., Sharma, N., Guo, V., Gong, Y., Anleu Gil, M.X., Weimer, A.K., and Bergmann, D.C. Single-cell resolution of lineage trajectories in the Arabidopsis stomatal lineage and developing leaf. *Dev. Cell* 56, 1043-1055.e4 (2021).
9. Zhang, T.Q., Chen, Y., and Wang, J.W. A single-cell analysis of the *Arabidopsis* vegetative shoot apex. *Dev Cell.* 56, 1056-1074.e8 (2021).
10. Martín, G., and Duque, P. Tailoring photomorphogenic markers to organ growth dynamics. *Plant Physiol.* 186, 239–249 (2021).

11. Kubo, M., Udagawa, M., Nishikubo, N., Horiguchi, G., Yamaguchi, M., Ito, J., Mimura, T., Fukuda, H., and Demura, T. Transcription switches for protoxylem and metaxylem vessel formation. *Genes Dev.* 19, 1855–1860 (2005).

12. DeWitt, N.D. and Sussman, M.R. Immunocytological localization of an epitope-tagged plasma membrane proton pump (H(+)-ATPase) in phloem companion cells. Plant Cell 7(12):2053-67 (1995).

13. Froelich, D.R., Mullendore, D.L., Jensen, K.H., Ross-Elliott, T.J., Anstead, J.A., Thompson, G.A., Pélissier, H.C., and Knoblauch, M. Phloem Ultrastructure and Pressure Flow: Sieve-Element-Occlusion-Related Agglomerations Do Not Affect Translocation. *Plant Cell* 23, 4428–4445 (2011).

14. Truernit, E., Bauby, H., Belcram, K., Barthélémy, J., and Palauqui, J.-C. OCTOPUS, a polarly localised membrane-associated protein, regulates phloem differentiation entry in *Arabidopsis thaliana*. *Development* 139, 1306–1315 (2012).

15. Kondo, T., Kajita, R., Miyazaki, A., Hokoyama, M., Nakamura-Miura, T., Mizuno, S., Masuda, Y., Irie, K., Tanaka, Y., Takada, S., et al. Stomatal Density is Controlled by a Mesophyll-Derived Signaling Molecule. *Plant Cell Physiol.* 51, 1–8 (2010).

16. Sessions, A., Weigel, D., and Yanofsky, M.F. (2002). The *Arabidopsis thaliana MERISTEM LAYER 1* promoter specifies epidermal expression in meristems and young primordia: Epidermal expression of transgenes in meristems. *Plant J.* 20, 259–263 (2002).

17. Aida, M., Ishida, T., and Tasaka, M. Shoot meristem and cotyledon formation. *Development* 126, 1563-1570 (1999).

18. Butler, A., Hoffman, P., Smibert, P., Papalexi, E., Satija, R. Integrating single-cell transcriptomic data across different conditions, technologies, and species. *Nat Biotechnol.* 36(5):411-420 (2018).

19. Wendrich, J.R., Yang, B., Vandamme, N., Verstaen, K., Smet, W., Van de Velde, C., Minne, M., Wybouw, B., Mor, E., Arents, H.E., et al. Vascular transcription factors guide plant epidermal responses to limiting phosphate conditions. *Science*.370(6518):eaay4970 (2020).

Supplementary Figures:

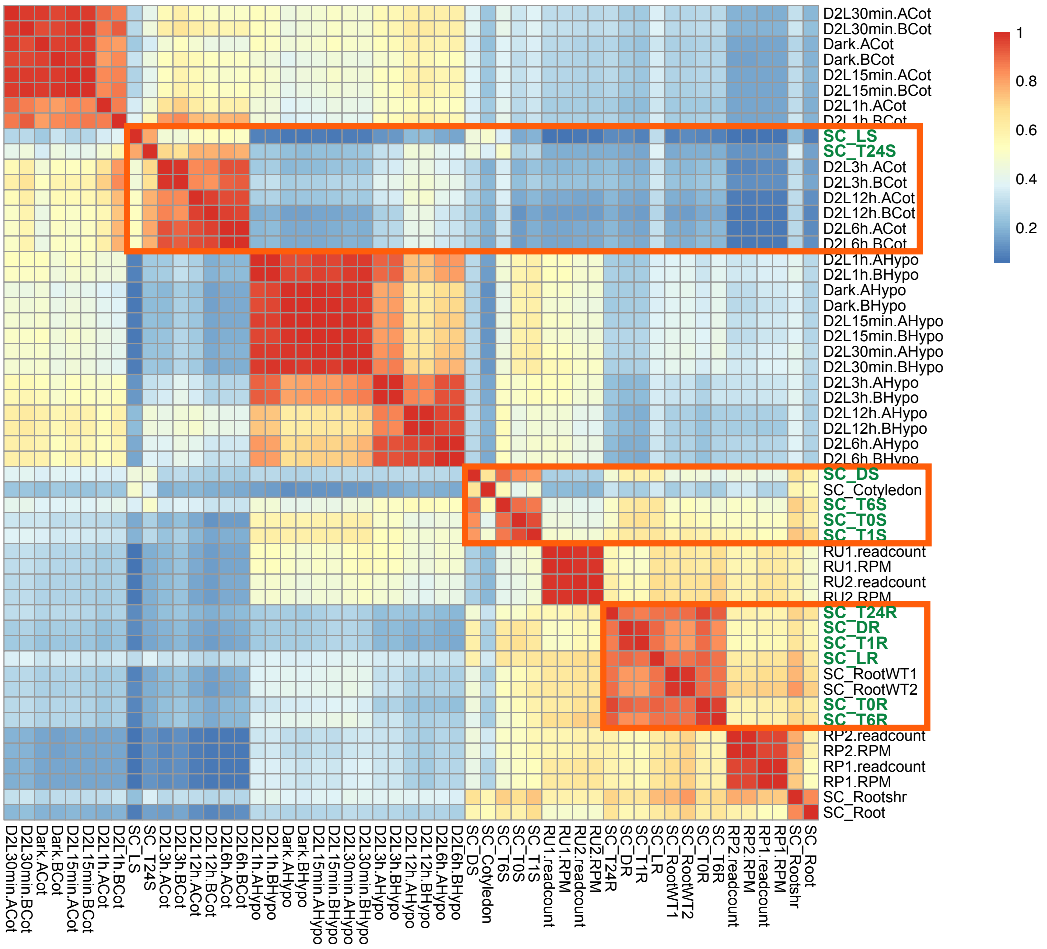

Figure S1. Correlation Coefficient of RNA-Seq Data.

The relative expression of bulk RNA-seq data and scRNA-seq data were merged together. Pearson correlation coefficients were calculated by the expression matrix. Color bar: correlation coefficient with whole genome expression levels.

SC: single cell RNA-seq data; SC_RootWT1[3], SC_RootWT2[3] and SC_Rootshr[3], SC_Root[4], SC_Cotyledon[5], and single cell samples from this study, SC_DS, SC_T0S, SC_T1S, SC_T6S, SC_T24S, SC_LS, SC_DR, SC_T0R, SC_T1R, SC_T6R, SC_T24R, SC_LR, which were colored by green for highlight.

RU[3] and RP[3]: bulk RNA-seq data for root; D2L[6] and Dark[6]: bulk RNA-Seq data for hypocotyl and cotyledon during de-etiolation.

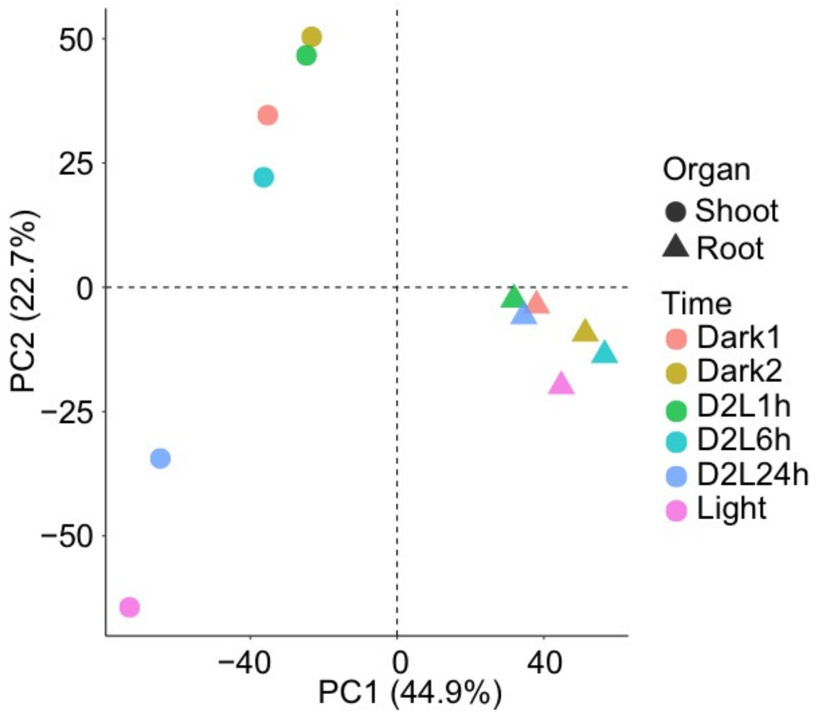

Figure S2. The Principal Component Analysis of ScRNA-Seq during De-Etiolation.
The relative expression values for scRNA-seq data were merged. Shape: different parts; Color: different sampling time points.

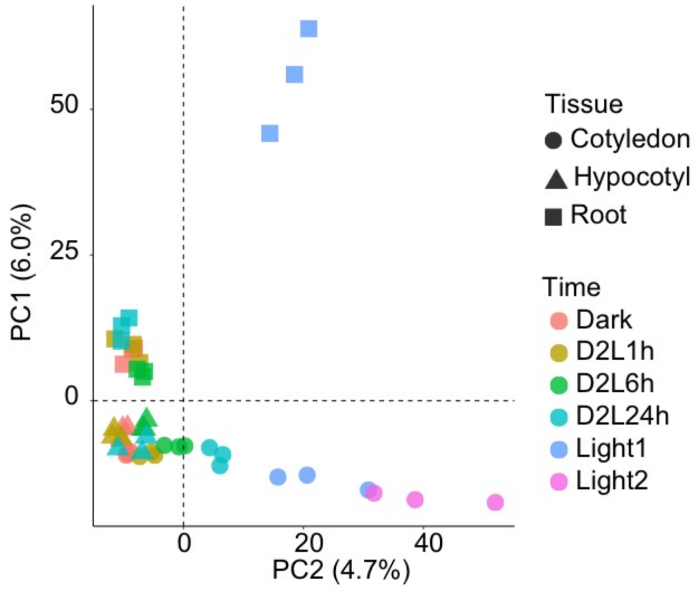

Figure S3. The Principal Component Analysis of Bulk RNA-Seq for Three Organs during De-Etiolation.
The FPKM values for bulk RNA-seq data were merged. Top 3000 genes with higher variances among samples were used for downstream analyses. Shape: different organs; Color: different sampling time points.

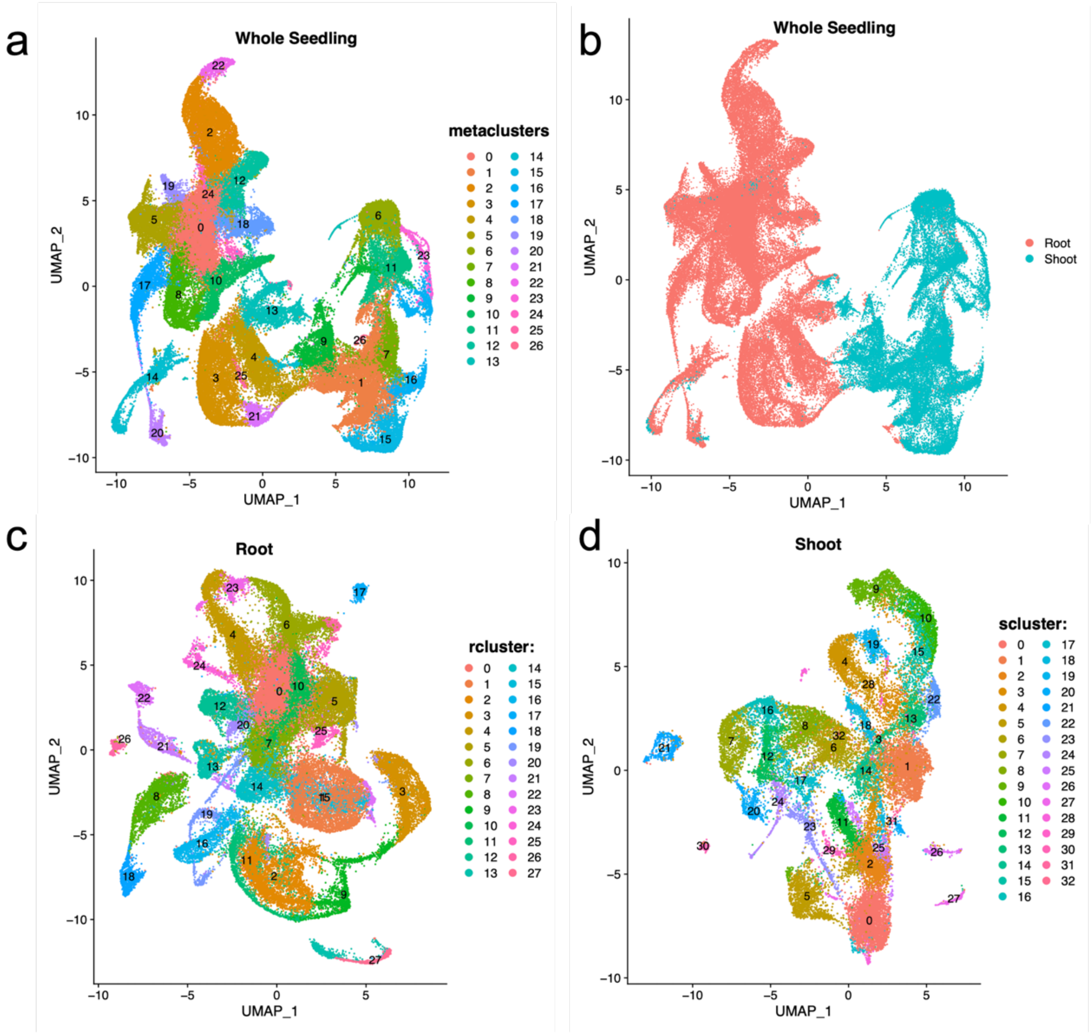

Figure S4. Visualization of Seedling Cells Using UMAP.

(a) Cell atlas constructed with merged shoot and root cells. Dot indicated individual cells while color represented respective metaclusters.

(b) The same atlas as (a) but classified by different tissues. Root (pink) and shoot (blue) cells were separated by left and right part by the UMAP coordinates.

(c) Root atlas with merged root cells (corresponding to Figure 1d). Cells with different colors indicated respective rclusters.

(d) Shoot atlas with merged shoot cells (corresponding to Figure 1a). Cells with different colors indicated respective sclusters.

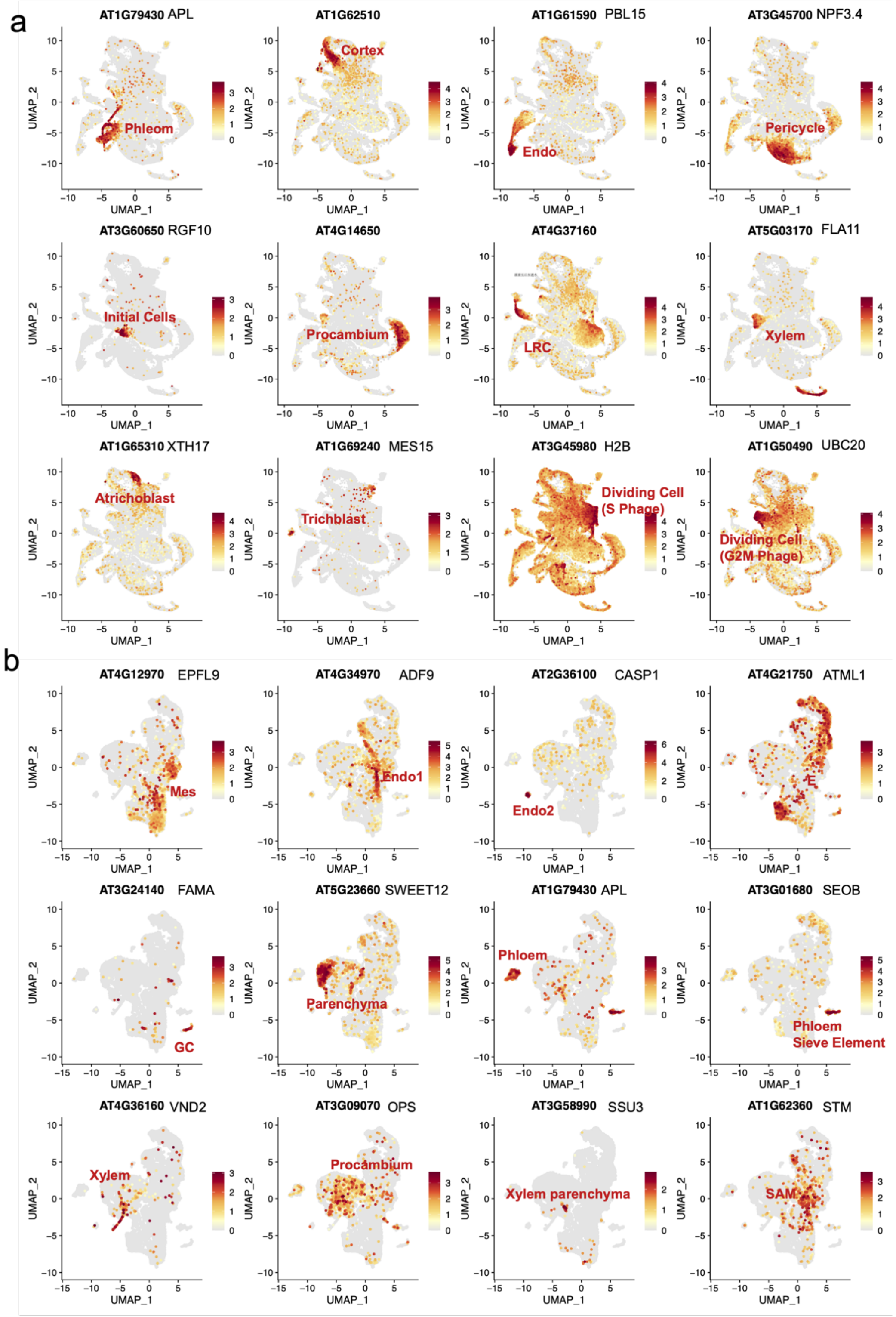

Figure S5. Expression Patterns of Cell Markers on the De-Etiolating Atlases.

(a) Expression of markers for annotation of root cell clusters.

(b) Expression of markers for annotation of shoot cell cluster.

Color: Normalized UMI counts. The cell type with enrichment of marker expression has been labeled beside.

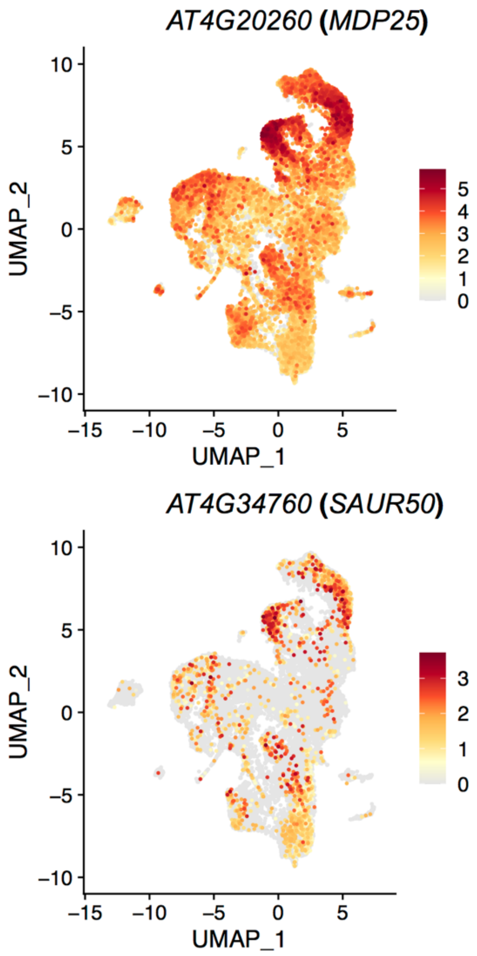

Figure S6. Expression Patterns of Hypocotyl Markers in Shoot Atlas.
The expression of hypocotyl higher expression gene *MDP25*, *HIPP31* and *SAUR50*. *HIPP31*[10] was specifically detected in hypocotyl by bulk RNA-seq data, *MDP25* and *SAUR50* showed higher expression in etiolated hypocotyl cells. Color: Normalized UMI counts.

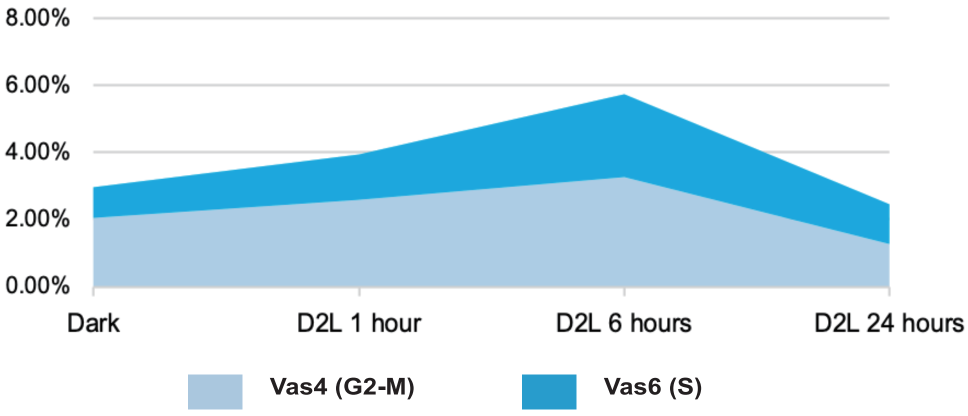

Figure S7. Proportion of Dividing Cells in De-Etiolation Process.
Cell proportions (cell number in Vas4 or Vas6/total cell number) for each sample have been calculated. Different cell types were colored by light and dark blue.

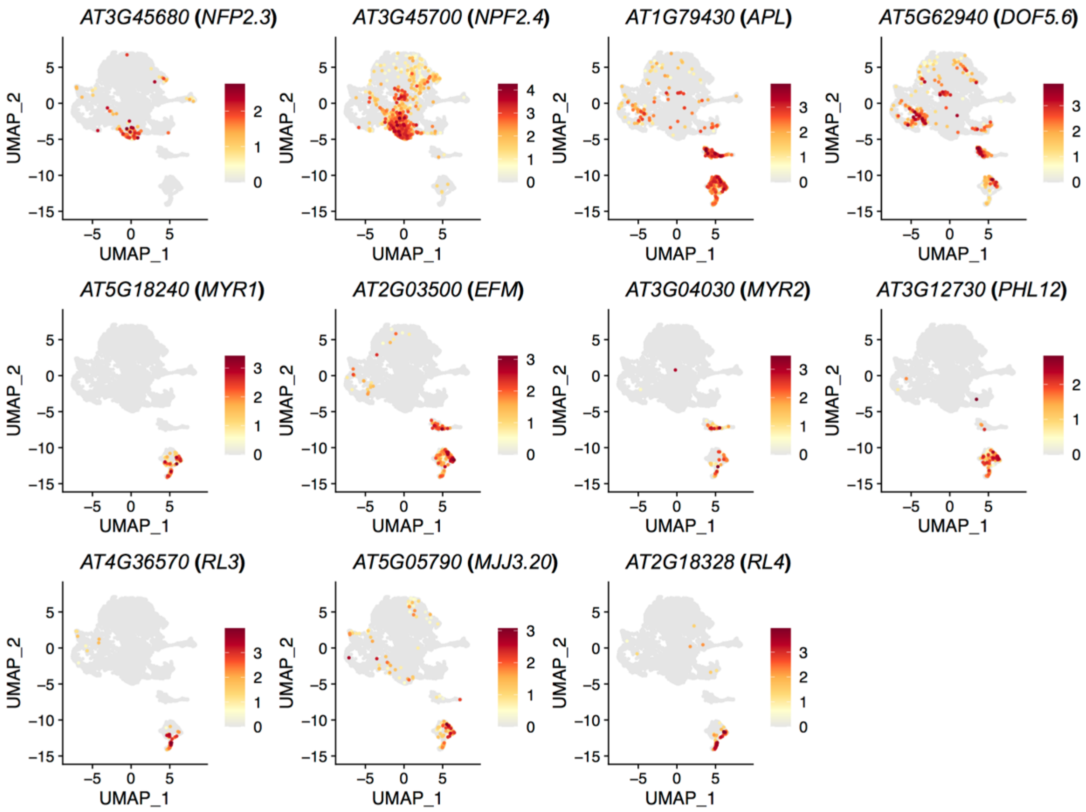

Figure S8. Expression Patterns of Candidate Vasculature Development Regulators in Vascular Cells.
Relative expression of candidate regulators in the shoot vasculature atlas. Color: Normalized UMI counts.

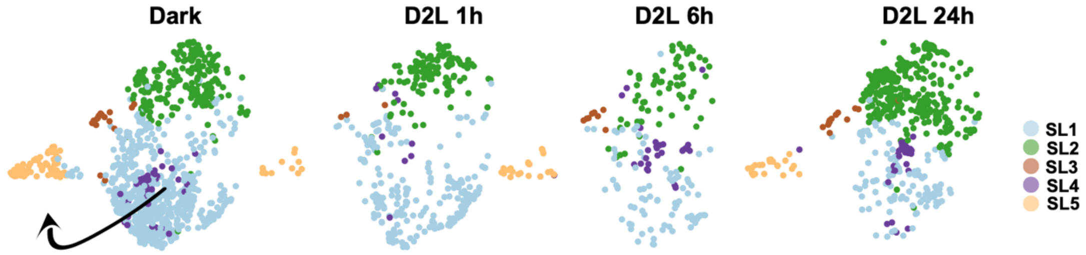

Figure S9. Visualization of SL Cells during the De-Etiolation Process.
SL cells during de-etiolation were embedded by UMAP. Dot in different colors indicated cells of different subtypes. The atlases were split by de-etiolation time points. The black arrow indicated canonical and dark-specific trajectories for GC development.

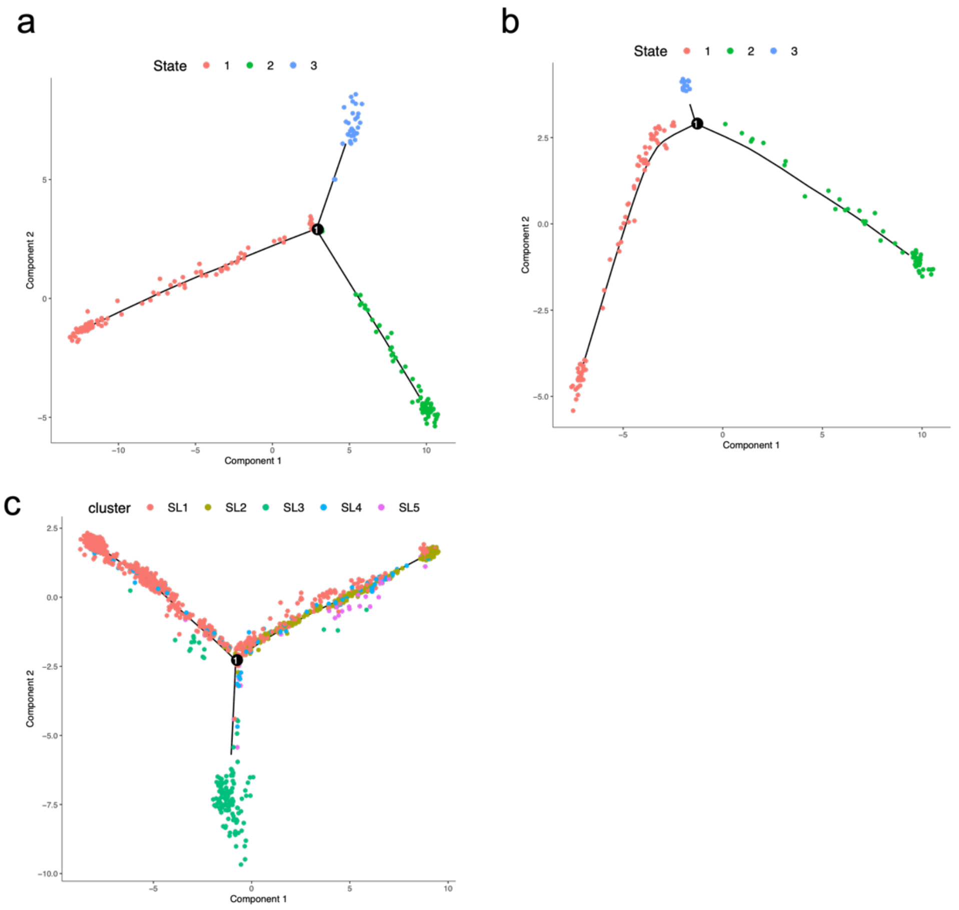

Figure S10. Trajectories of GCs.
(a) Development states of SL cells in canonical light trajectories. Cells were colored by different states (related to Figure 5g).
(b) Development states of SL cells in dark-specific trajectories. Cells were colored by different states (related to Figure 5g).

(c) Development states of SL cells merged from de-etiolation samples. Cells were colored by different subtypes.

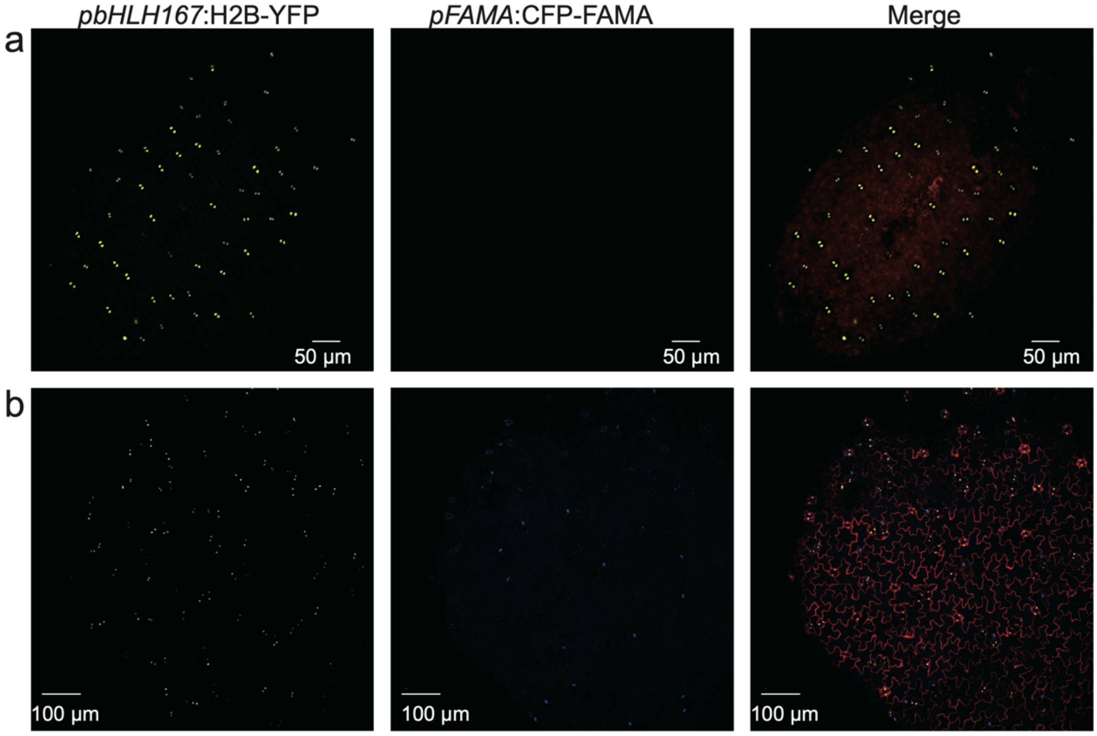

Figure S11. Expression of *bHLH167* in Epidermis for Seedlings Grown in Constant Dark for 3 Days.

The gene expression pattern of *bHLH167* and *FAMA* was revealed by promoter Histone2B-YFP (H2B-YFP, yellow) and CFP-FAMA (blue) reporter. The cell outline (red) was visualized with the DsRed2 reporter drived by a seedling specific promoter (*pAT2S3*). Each experiment was repeated for three times independently.

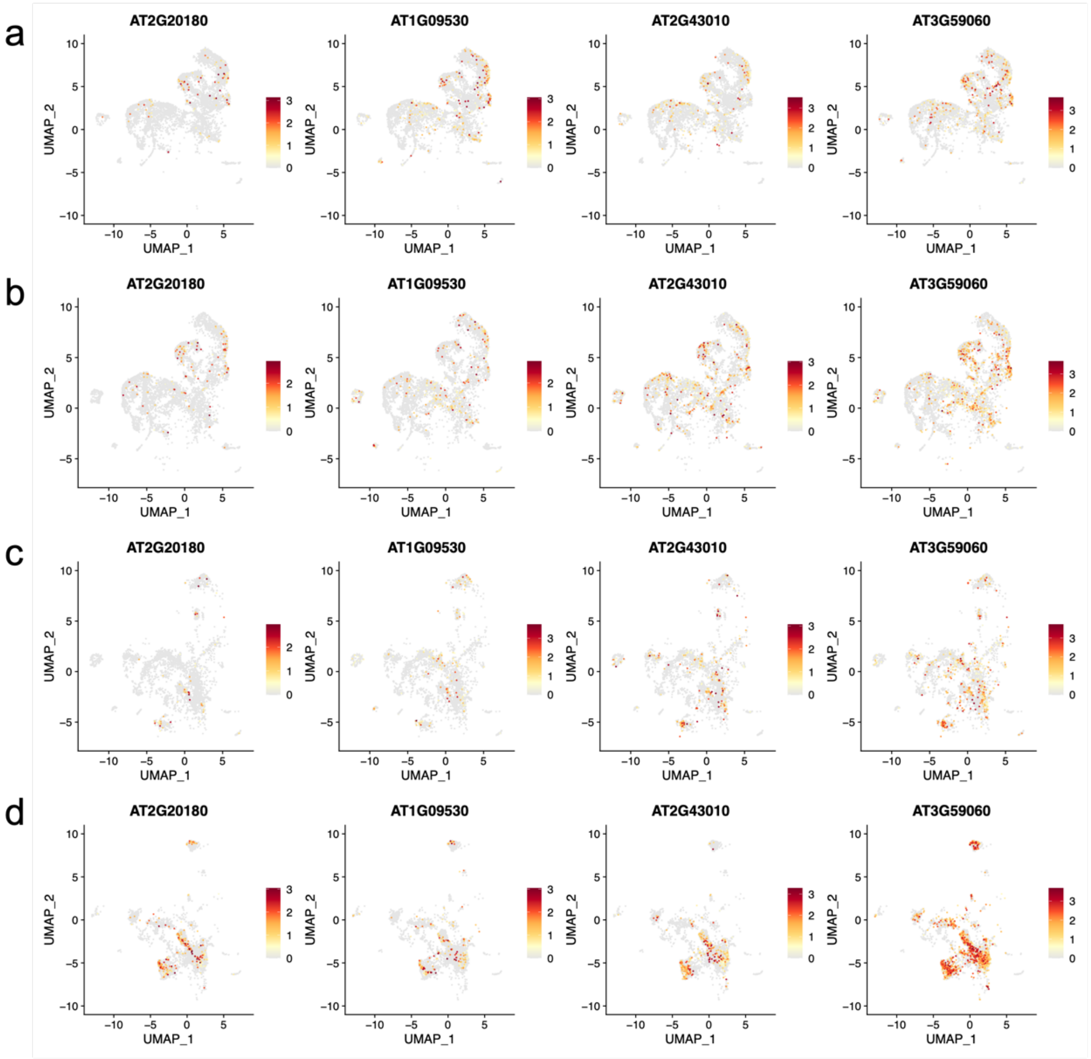

Figure S12. Expression Patterns of PIFs.

(a) The relative expression of *PIF1*, *PIF3*, *PIF4* and *PIF5* in Dark sample of shoot atlas.

(b) The relative expression of *PIF1*, *PIF3*, *PIF4* and *PIF5* in D2L1h sample of shoot atlas.

(c) The relative expression of *PIF1*, *PIF3*, *PIF4* and *PIF5* in D2L6h sample of shoot atlas.

(d) The relative expression of *PIF1*, *PIF3*, *PIF4* and *PIF5* in Light sample of shoot atlas.

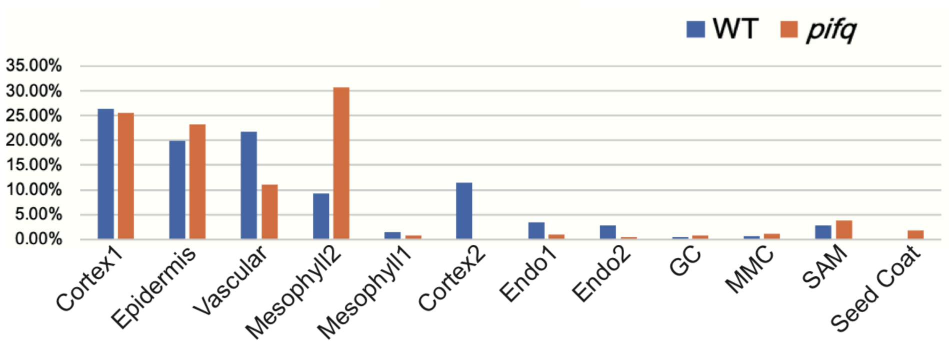

Figure S13 Cell Proportions for Dark-Grown WT and *pifq* Shoot.
Cell proportions (cell number for one cell type/ total cell number in the atlas) for each cell type in dark-grown WT and the *pifq* mutant atlas.

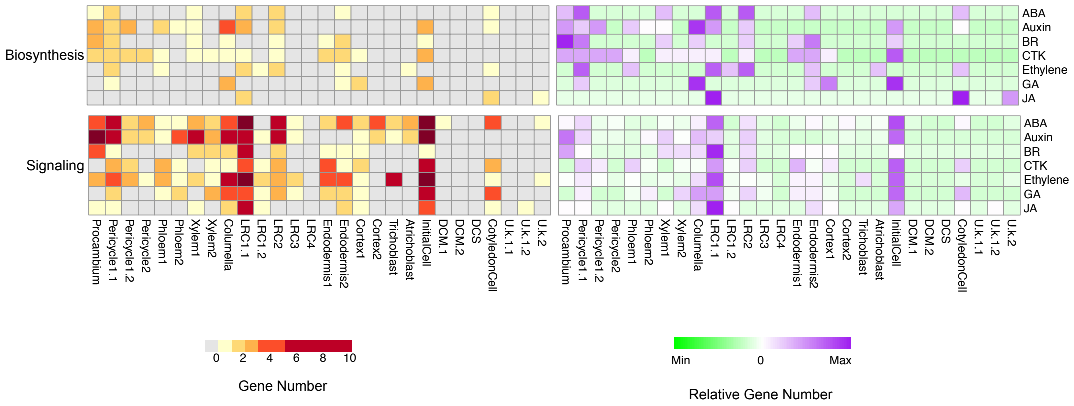

Figure S14 Distribution of Spatiotemporally Specific Genes Involved in Hormone Biosynthesis (Upper) and Signaling (Lower) in Root.
Left: Absolute value; Right: Relative value.

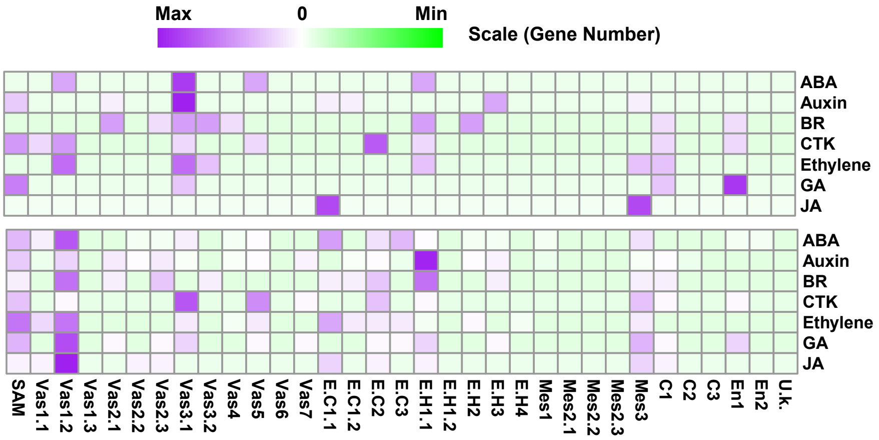

Figure S15 Distribution of Spatiotemporally Specific Genes Involved in Hormone Biosynthesis (Upper) and Signaling (Lower) in Shoot.

Color bar: scaled (number of genes with enriched expression in respective cell types) .

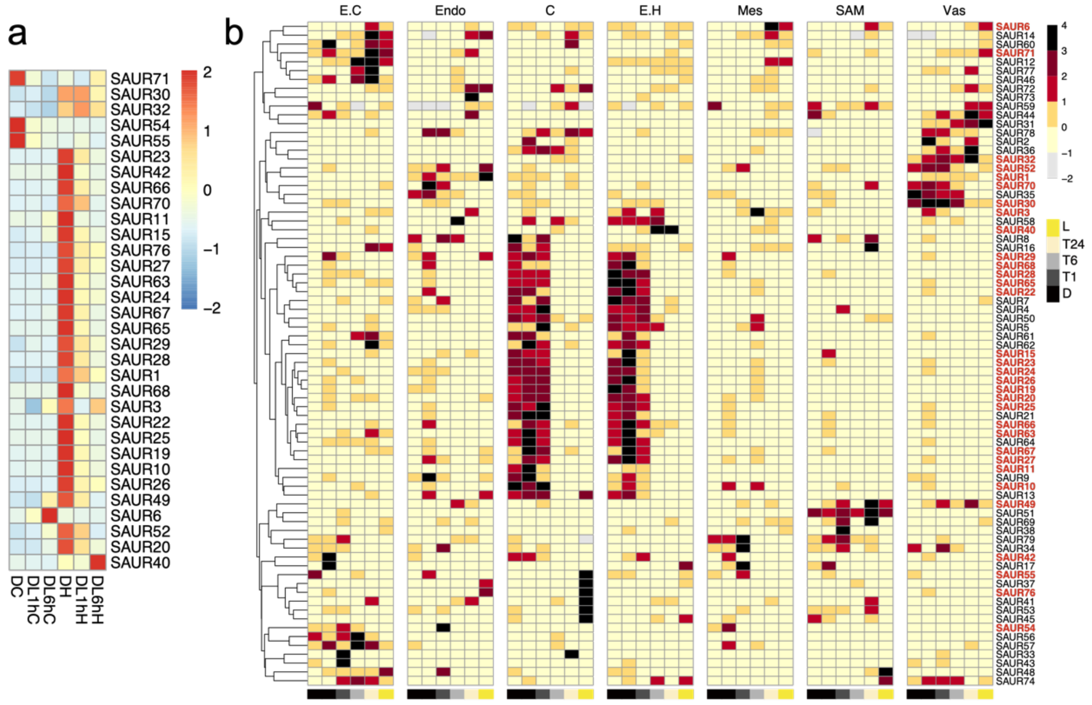

Figure S16. Spatiotemporal Expression Patterns of *SAUR* genes during De-Etiolation.

(a) Published expression patterns of *SAUR* genes. Each line indicated a *SAUR* gene. Darker color indicated higher expression levels. D: Constant dark; DL1h: Exposed to light for 1 hour after constant dark treatment; DL6h: Exposed to light for 6 hours after constant dark treatment; C: Cotyledon; H: Hypocotyl.

(b) Expression patterns of *SAUR* genes detected in this study. Each line indicated a *SAUR* gene. Darker color indicated higher expression levels. D: Constant dark; T1: Exposed to light for 1 hour after constant dark treatment; T6: Exposed to light for 6

hours after constant dark treatment; T24: Exposed to light for 24 hours after constant dark treatment; L: Constant light.

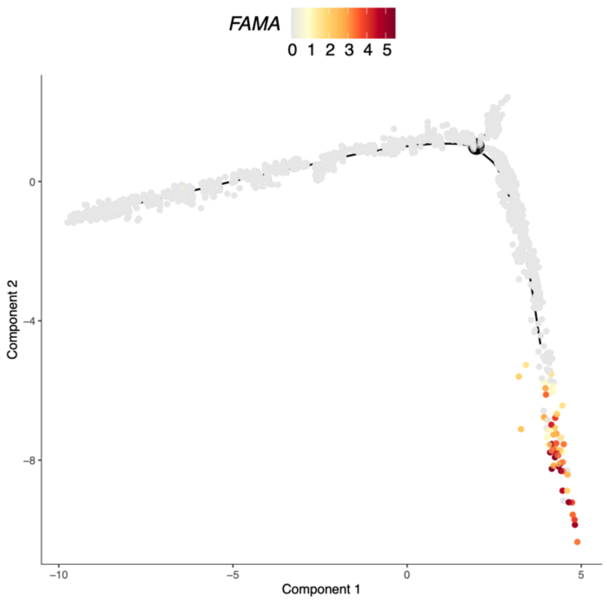

Figure S17. The Development Trajectory of Epidermal Cells for Dark-Grown WT and the *pifq* Mutant.

Expression levels of *FAMA* in each cell have indicated by different colors.

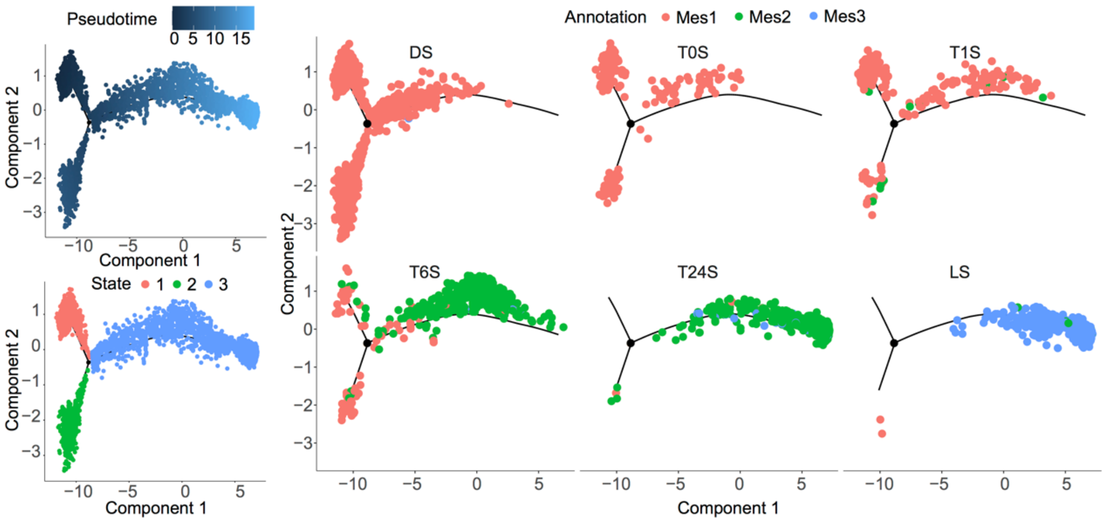

Figure S18. The Development Trajectories of Mesophyll Cells during De-Etiolation.

The development trajectory of mesophyll cells for de-etiolating samples. Pseudotime and cell states inferred by expression patterns have been indicated in the left panels. Cell types were highlighted by different colors in respective samples in right panels. DS and T0S represented two replicates of Dark samples.

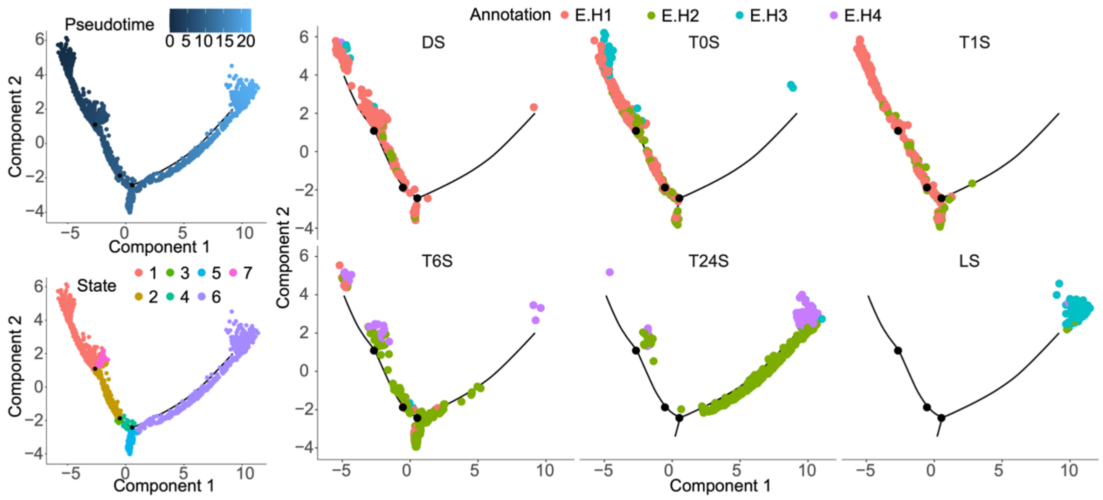

Figure S19. The Development Trajectories of Hypocotyl Epidermal Cells during De-Etiolation.

The development trajectory of E.H cells for de-etiolating samples. Pseudotime and cell states inferred by expression patterns have been indicated in the left panels. Cell types were highlighted by different colors in respective samples in right panels. DS and T0S represented two replicates of Dark samples.

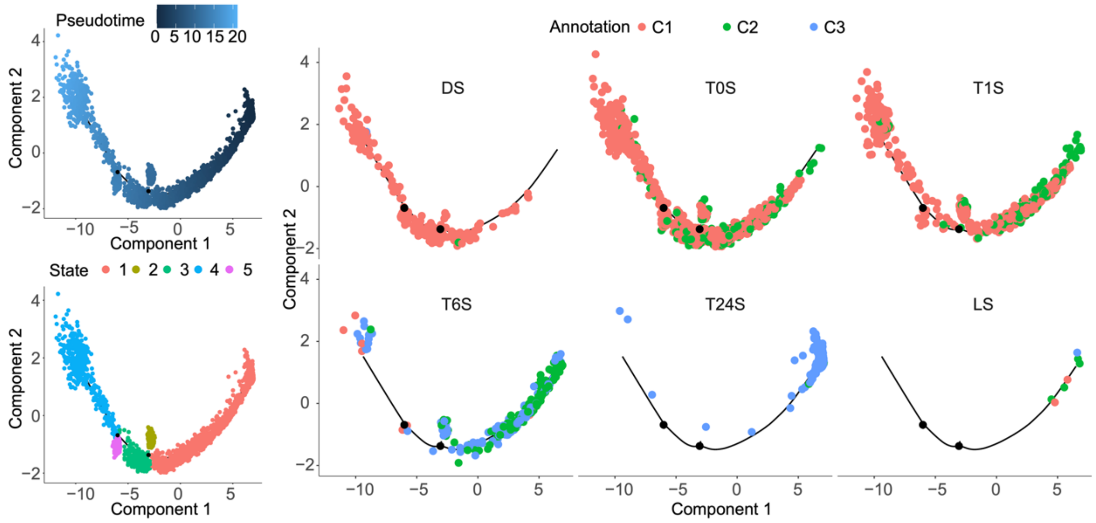

Figure S20. The Development Trajectories of Cortex Cells during De-Etiolation.
The development trajectory of cortical cells for de-etiolating samples. Pseudotime and cell states inferred by expression patterns have been indicated in the left panels. Cell types were highlighted by different colors in respective samples in right panels. DS and T0S represented two replicates of Dark samples.

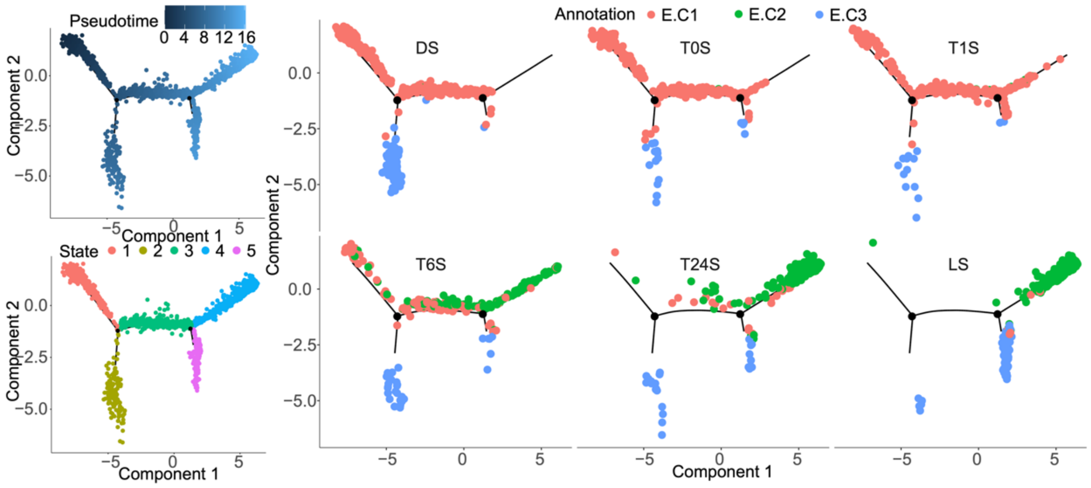

Figure S21. The Development Trajectories of Cotyledon Epidermal Cells during De-Etiolation.
The development trajectory of E.C cells for de-etiolating samples. Pseudotime and cell states inferred by expression patterns have been indicated in the left panels. Cell types were highlighted by different colors in respective samples in right panels. DS and T0S represented two replicates of Dark samples.

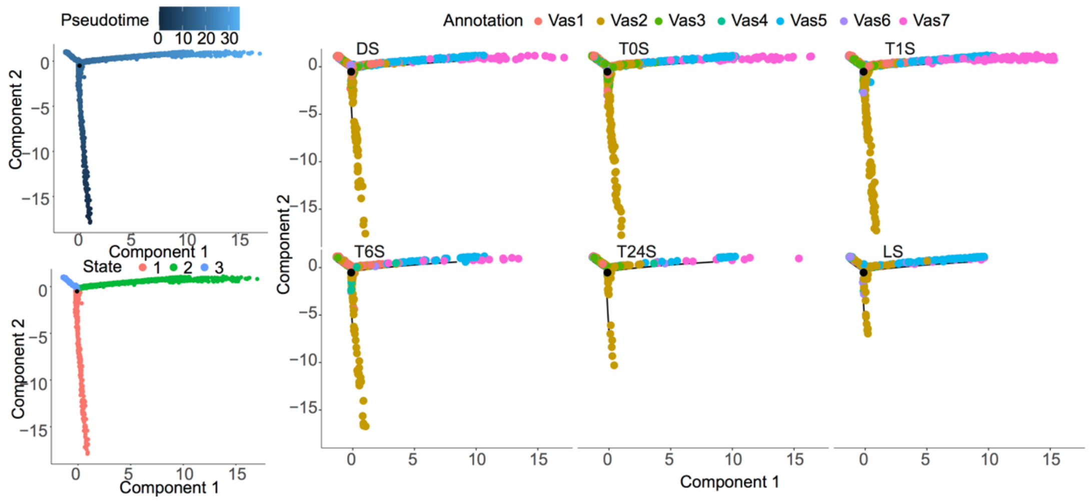

Figure S22. The Development Trajectories of Vascular Cells during De-Etiolation.
The development trajectory of vasculature cells for de-etiolating samples. Pseudotime and cell states inferred by expression patterns have been indicated in the left panels. Cell types were highlighted by different colors in respective samples in right panels. DS and T0S represented two replicates of Dark samples.

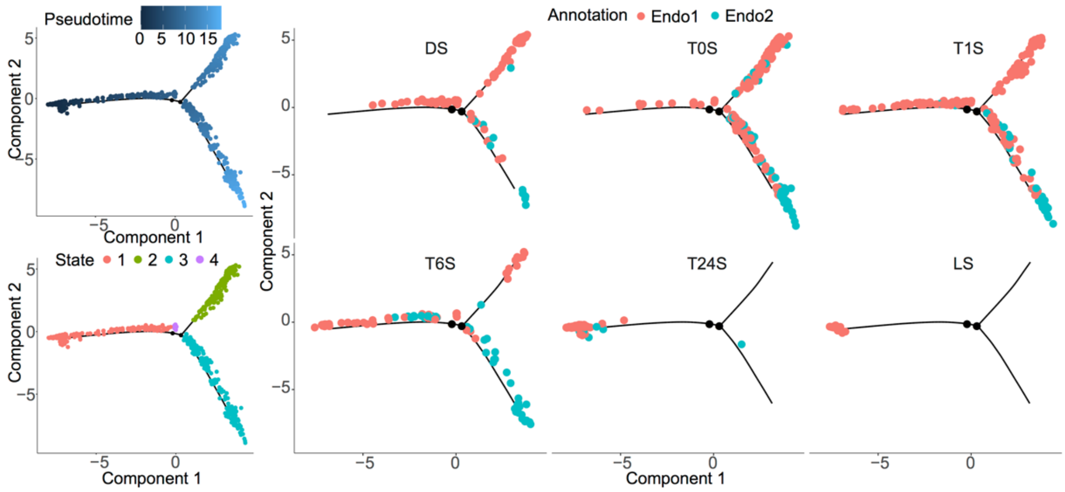

Figure S23. The Development Trajectories of Endodermis Cells during De-Etiolation.
The development trajectory of endodermal cells for de-etiolating samples. Pseudotime and cell states inferred by expression patterns have been indicated in the left panels. Cell types were highlighted by different colors in respective samples in right panels. DS and T0S represented two replicates of Dark samples.

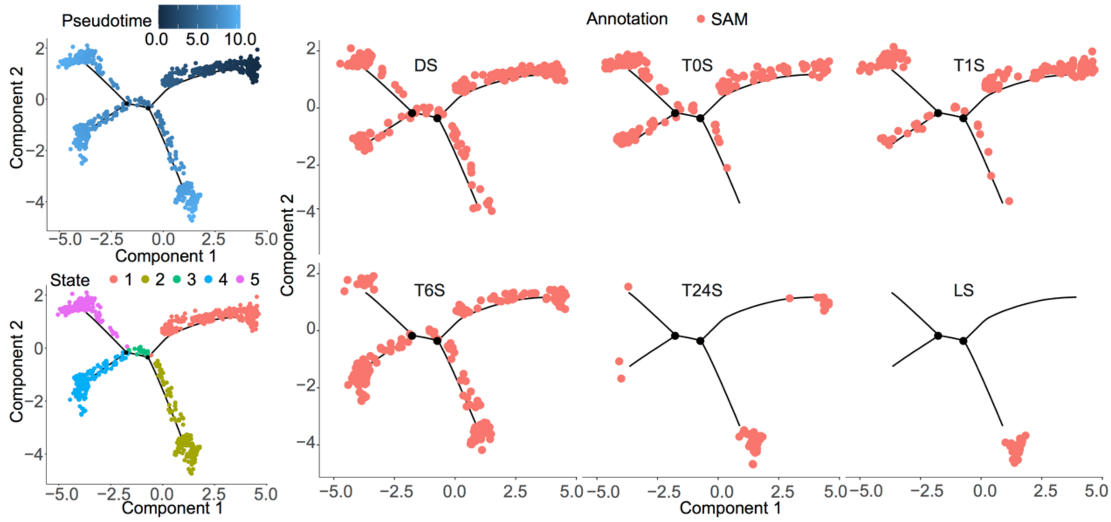

Figure S24. The development trajectories of SAM Cells during De-Etiolation.
The development trajectory of SAM cells for de-etiolating samples. Pseudotime and cell states inferred by expression patterns have been indicated in the left panels. Cell types were highlighted by different colors in respective samples in right panels. DS and T0S represented two replicates of Dark samples.

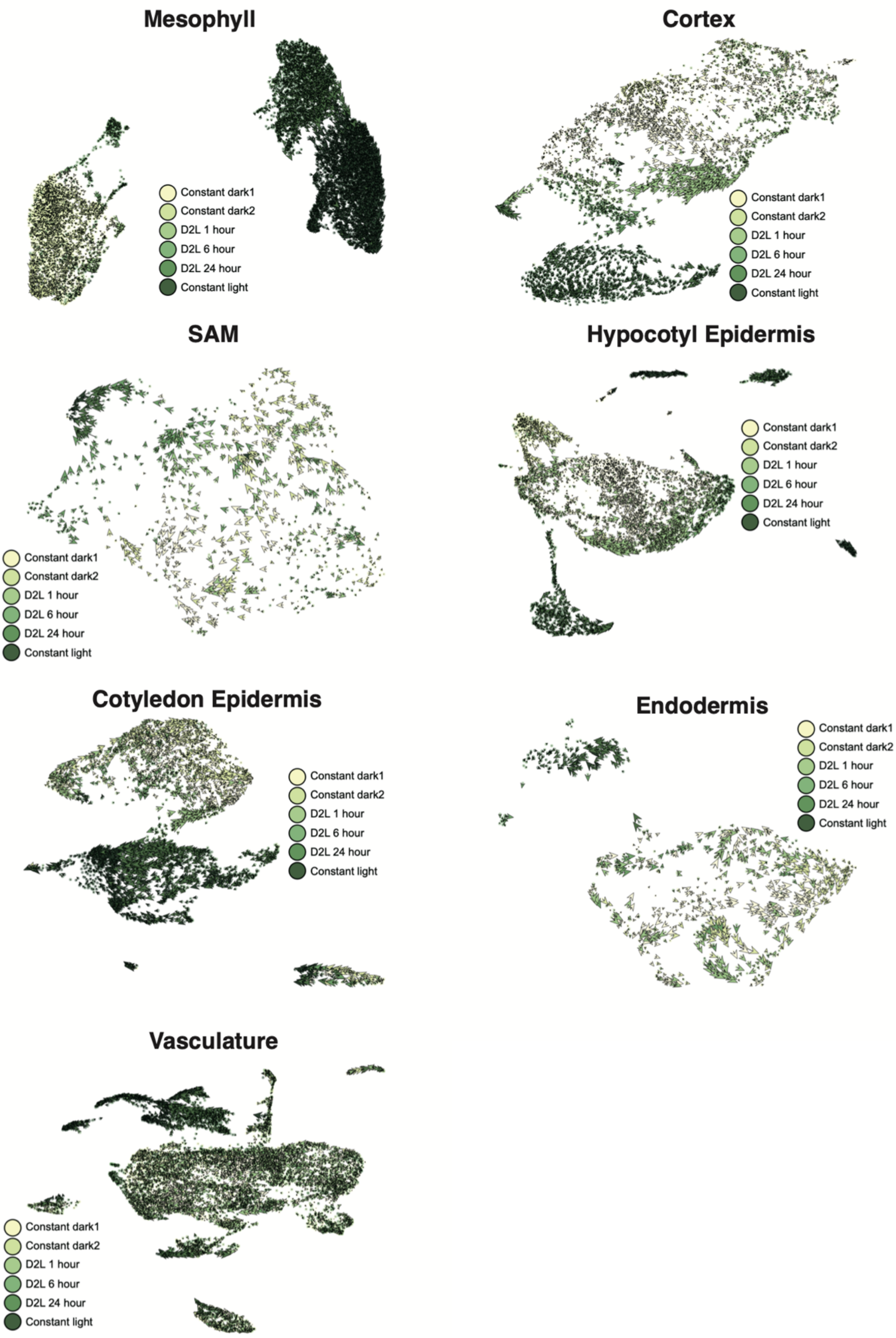

Figure S25. The Velocities for Seven Cell Types during De-Etiolation.

The cell atlas constructed for Mesophyll, Cortex, SAM, E.H, E.C, Endodermis, and Vasculature cells were viewed by UMAP. RNA velocities were inferred by ScVelo. The direction of arrow indicated the inferred development directions. Cells were colored by different samples.

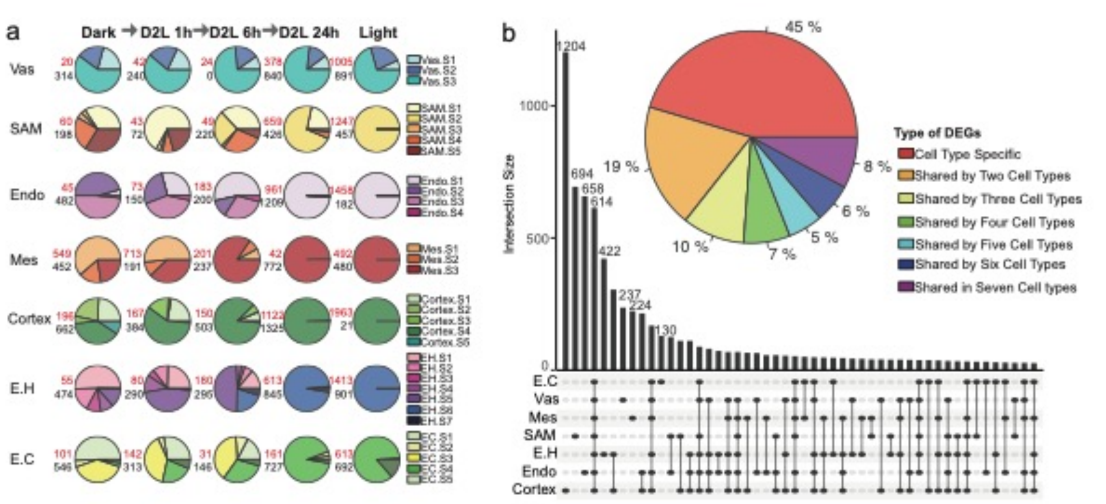

Figure S26 Light Responsive Genes Identified for Shoot Cell Types.

(a) Number of light responsive genes identified in respective cell types during the de-etiolation process. Red number indicated up-regulated genes, while black number indicated down-regulated gene. Cell proportions of different states identified by trajectory analyses were illustrated by pie charts.

(b) Distribution of light responsive genes detected in each shoot cell type.

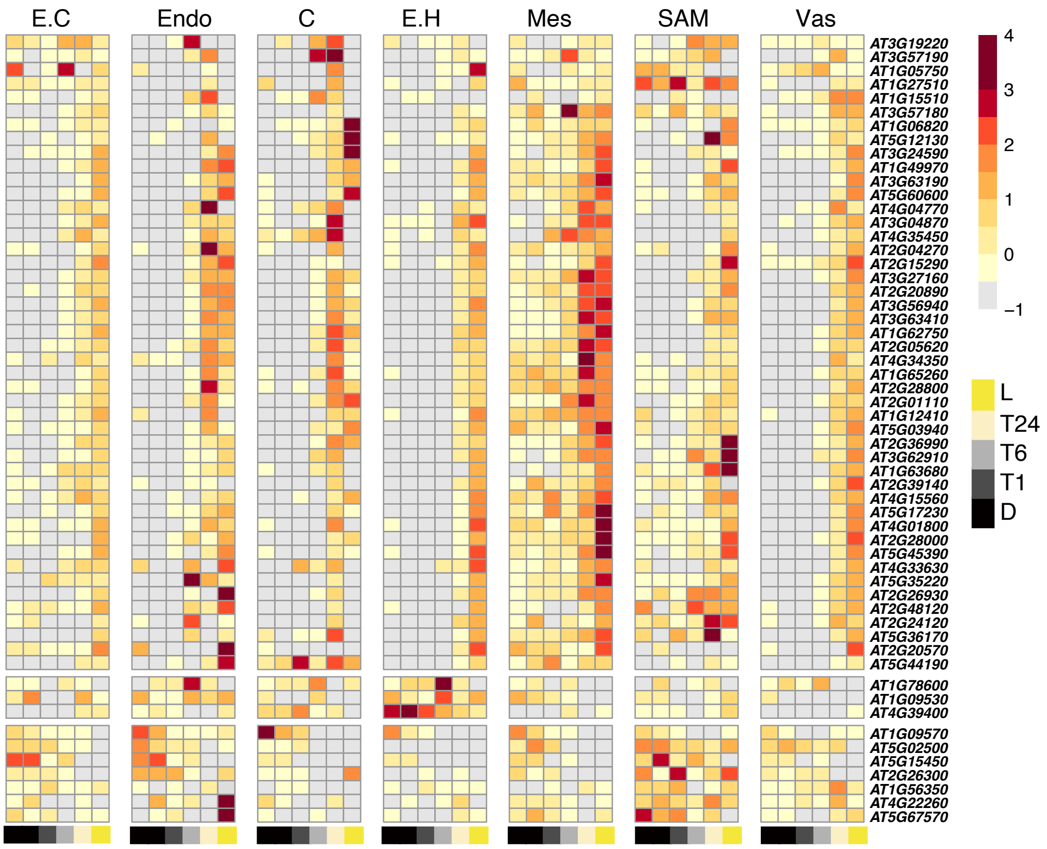

Figure S27 The expression patterns of gene set 1 involved in plastid biogenesis. Expression patterns of causal genes for abnormal phenotypes in chloroplast biogenesis and development identified in *Arabidopsis*. Each line indicated a gene. Darker color indicated higher expression levels. D: Constant dark; T1: Exposed to light for 1 hour after constant dark treatment; T6: Exposed to light for 6 hours after constant dark treatment; T24: Exposed to light for 24 hours after constant dark treatment; L: Constant light

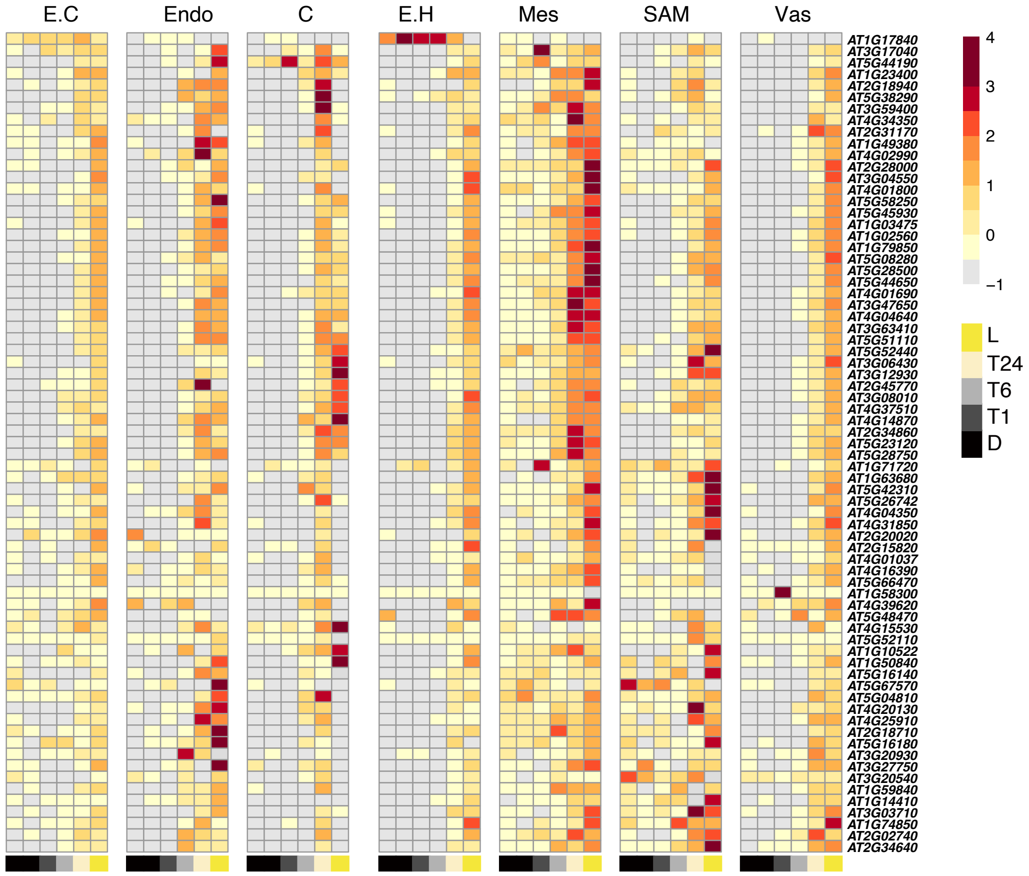

Figure S28 The expression patterns of gene set 2 involved in plastid biogenesis. Expression patterns of *Arabidopsis* orthologs of maize genes mapped with a photosynthetic mutant library. Each line indicated a gene. Darker color indicated higher expression levels. D: Constant dark; T1: Exposed to light for 1 hour after constant dark treatment; T6: Exposed to light for 6 hours after constant dark treatment; T24: Exposed to light for 24 hours after constant dark treatment; L: Constant light

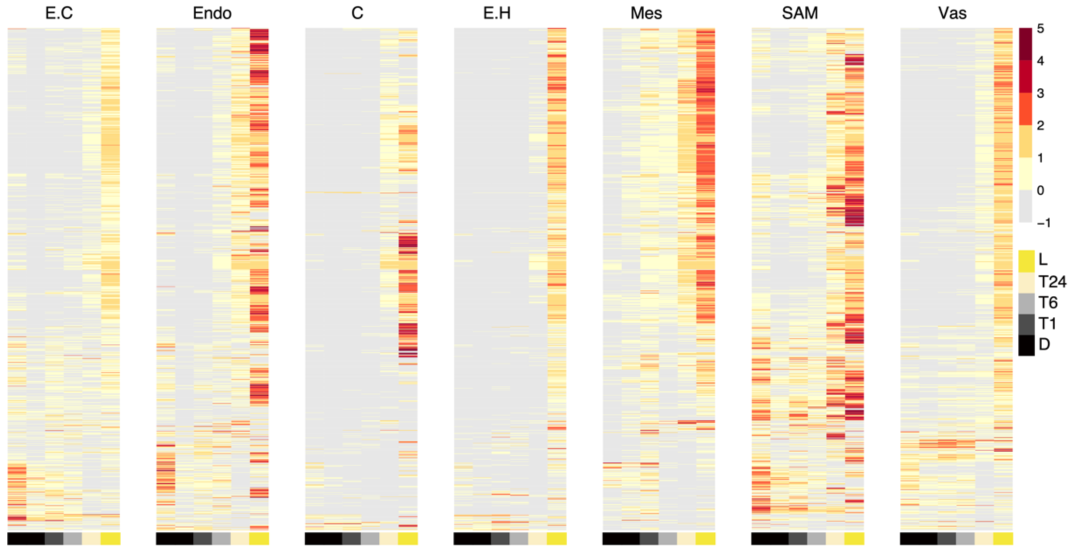

Figure S29 The Expression Patterns of Genes Involved in Plastid Biogenesis. Expression patterns of nuclear chloroplast biogenesis genes in shoot cell types during de-etiolation. Each line indicated a gene. Darker color indicated higher expression levels. D: Constant dark; T1: Exposed to light for 1 hour after constant dark treatment; T6: Exposed to light for 6 hours after constant dark treatment; T24: Exposed to light for 24 hours after constant dark treatment; L: Constant light

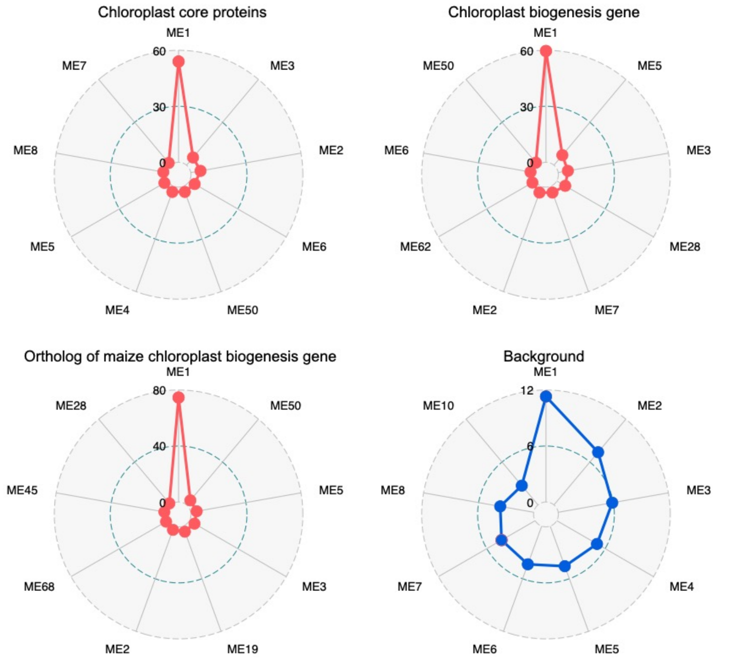

Figure S30 Radar plots showing the distribution of co-expression modules of chloroplast biogenesis genes.

The top 9 enriched modules have been illustrated. Numbers adjacent to circles showed the percentage of genes belong to respective modules. All genes used for the construction of spatiotemporal co-expression networks have been selected as background. The enrichment signals were significant in three candidate genes sets comparing to background.

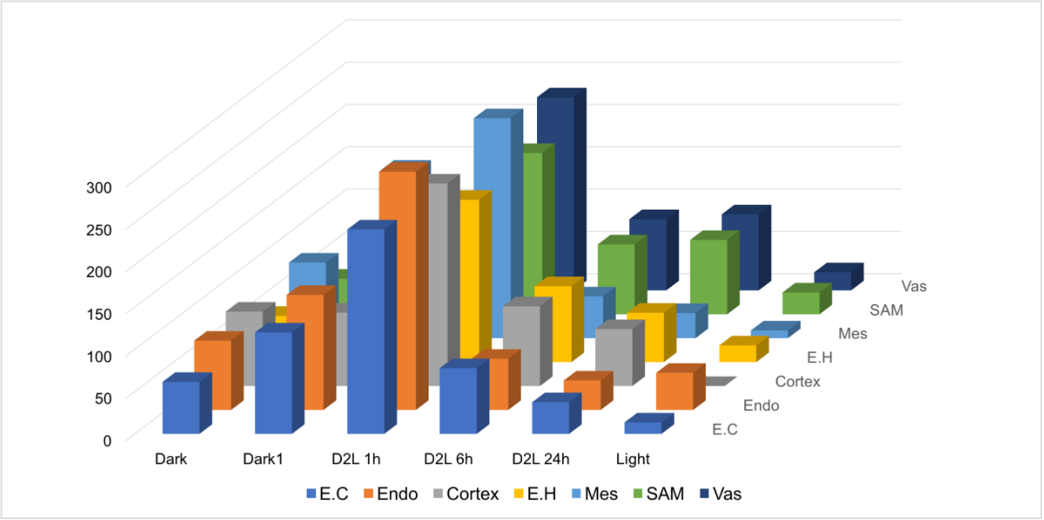

Figure S31. Relative Expression of *HY5* during De-Etiolation in Each Shoot Cell Type.

Mean of normalized UMI counts for each cell type have been calculated.

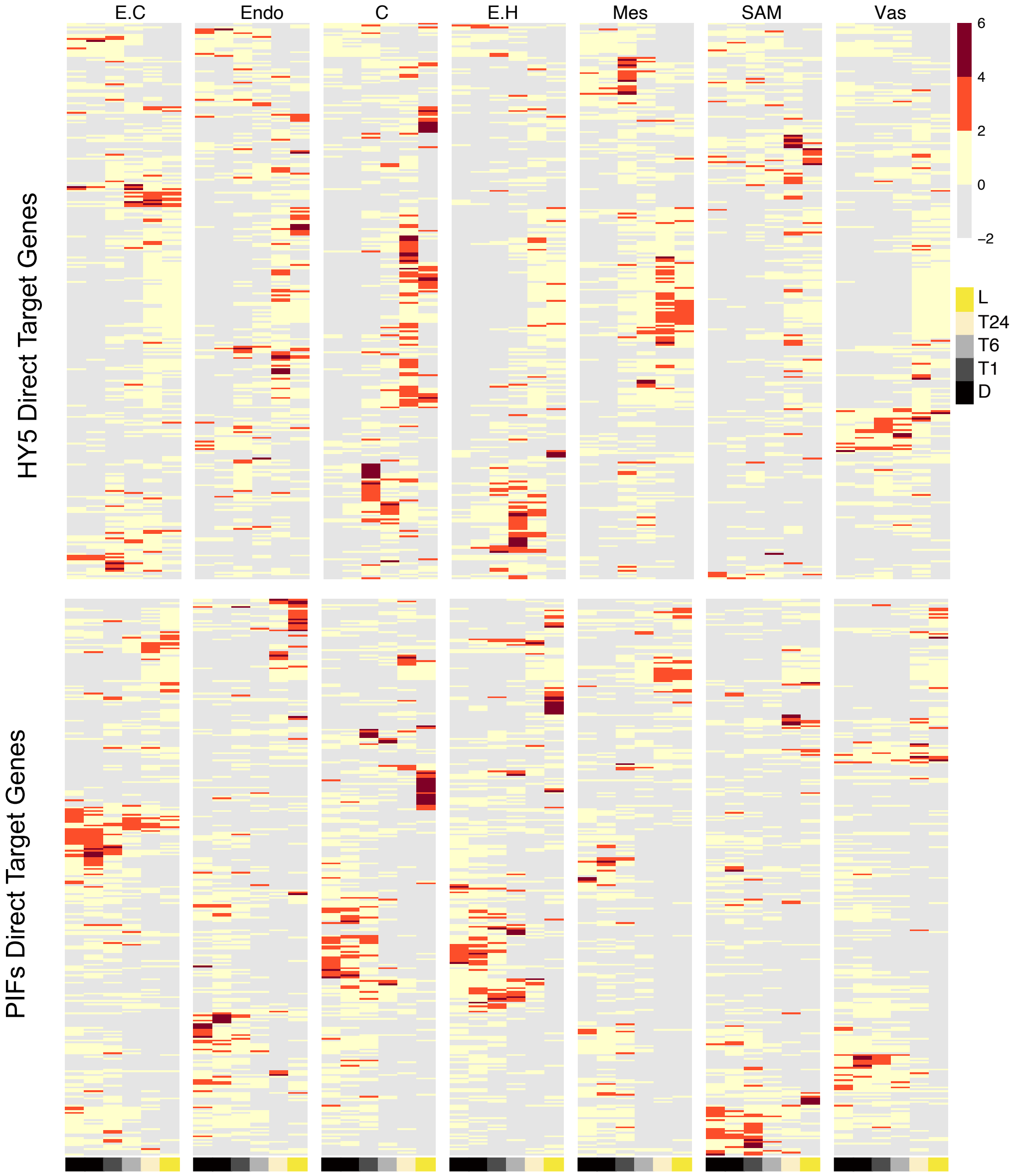

Figure S32. Relative expression for direct targe genes of HY5 and PIFs during de-etiolation.

Each line indicated a target gene. Darker color indicated higher expression levels. D: Constant dark; T1: Exposed to light for 1 hour after constant dark treatment; T6: Exposed to light for 6 hours after constant dark treatment; T24: Exposed to light for 24 hours after constant dark treatment; L: Constant light.

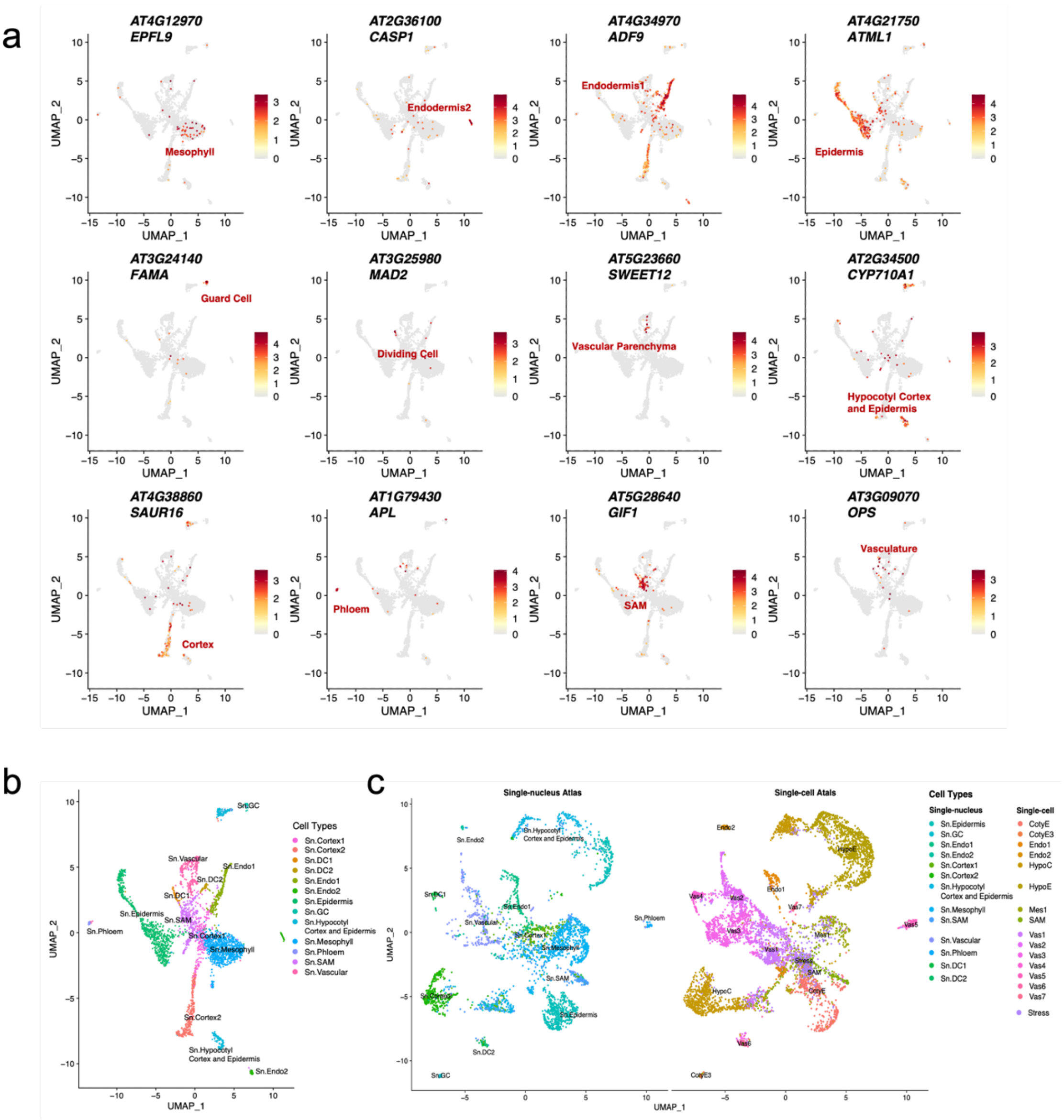

Figure S33. Expression Patterns of Shoot Cell Markers in Single Nucleus Atlas.
(a) Expression of markers for annotation of shoot atlas constructed with snRNA-seq data. The cell type with enrichment of marker expression has been labeled beside. Color: Normalized UMI counts.
(b) Visualization of shoot atlas of constructed with snRNA-seq data. Dots with different colors indicated different cell types. Cell type annotations have also been labeled.
(c) Visualization of shoot atlas of etiolated seedlings constructed by snRNA-seq data and scRNA-seq data. The snRNA-seq data were integrated with Dark data by integration pipeline for batch effect correction. Cell type annotations have been labeled beside respective cell types.

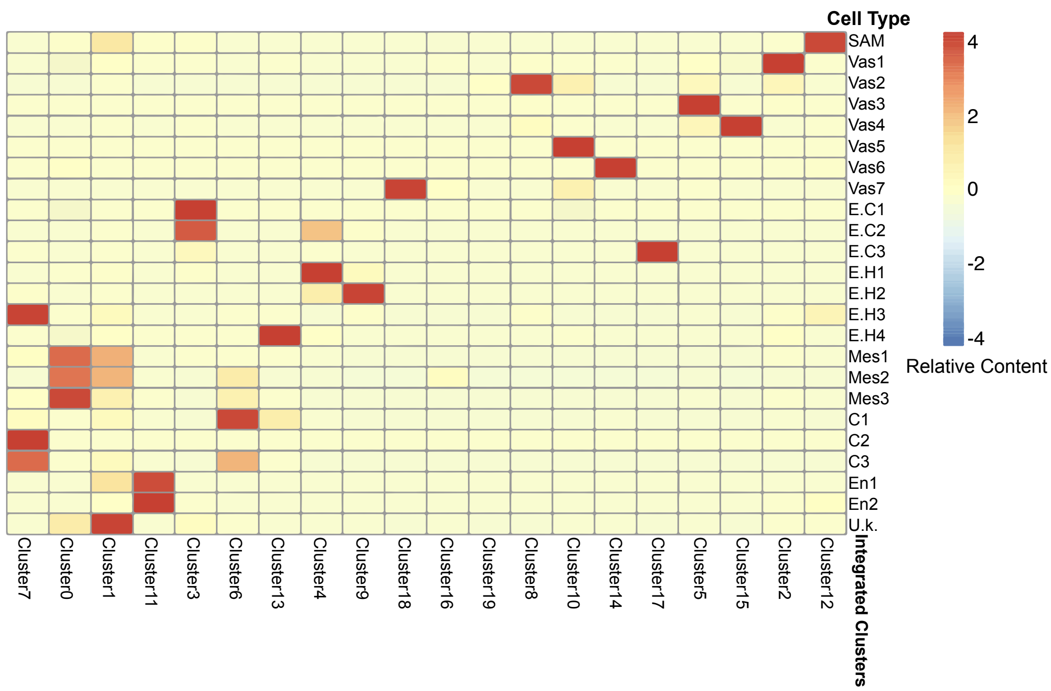

Figure S34. Cell Cluster Correspondence before and after Batch Effect Correction. Y axis indicated annotation of cell clusters in shoot atlas. X axis indicated cell clusters classified after batch effect correlation. The higher proportion of overlapped cells of a cell type (y-axis) and integrated clusters (x-axis) indicated the correspondence. For some cell types corresponding to the same integrated cluster indicated these cell types were same cells with transcriptome changes induced by light. Color: scaled (overlapped cell proportions).

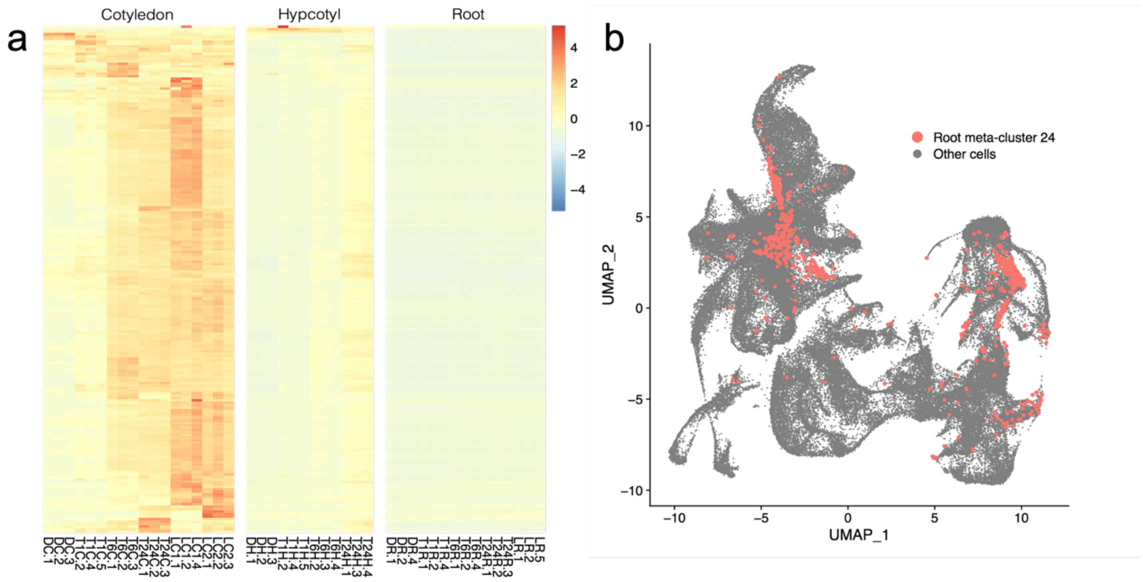

Figure S35. Annotation of Rcluster 24.

(a) The expression patterns of top 100 genes highly expressed in rcluster 24 identified by FindMarkers in organ-specific bulk RNA-seq data during de-etiolation. Significant expression enrichment has been detected in cotyledon cells.

(b) The distribution of cells from rcluster 24 in seedling atlas. Cells of rcluster 24 have been highlighted in pink.

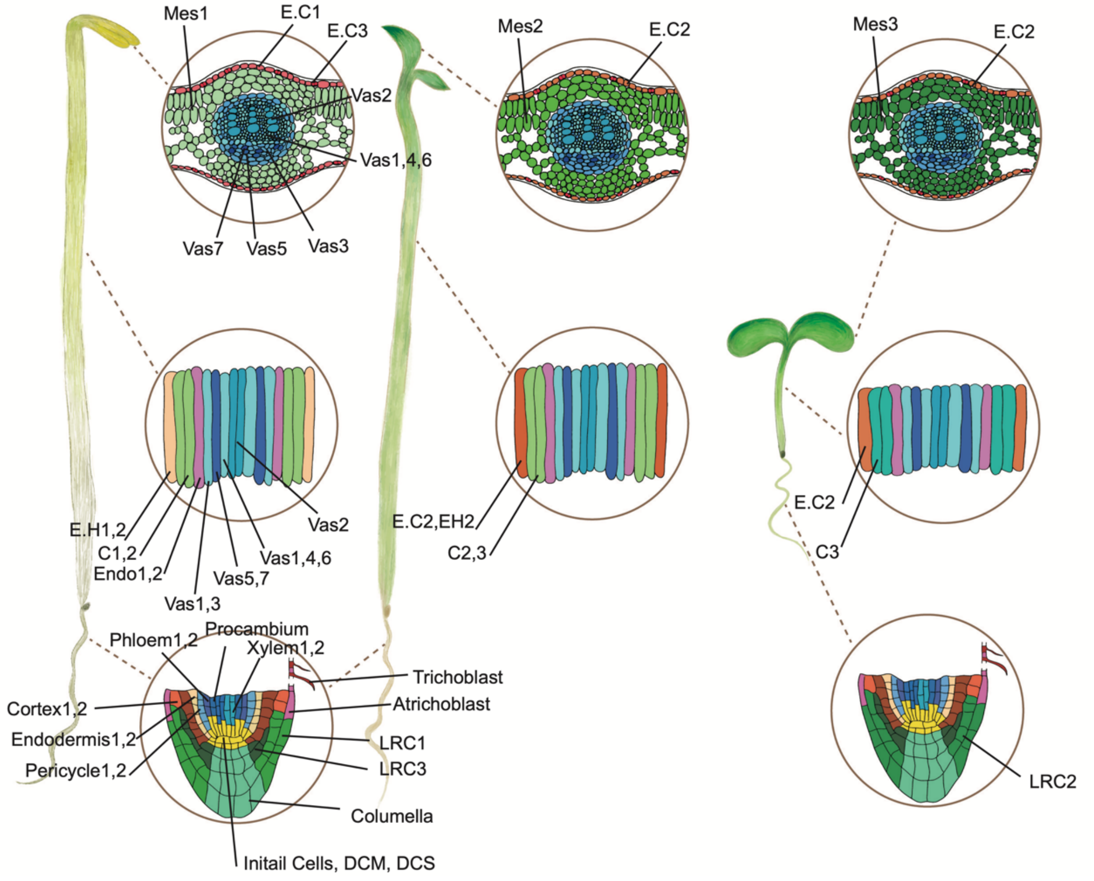

Figure S36. A diagram Indicating the Location of the Seedling for Respective Cell Types.

Different cell states for the same cell types were labeled in de-etiolated and light-grown seedlings.

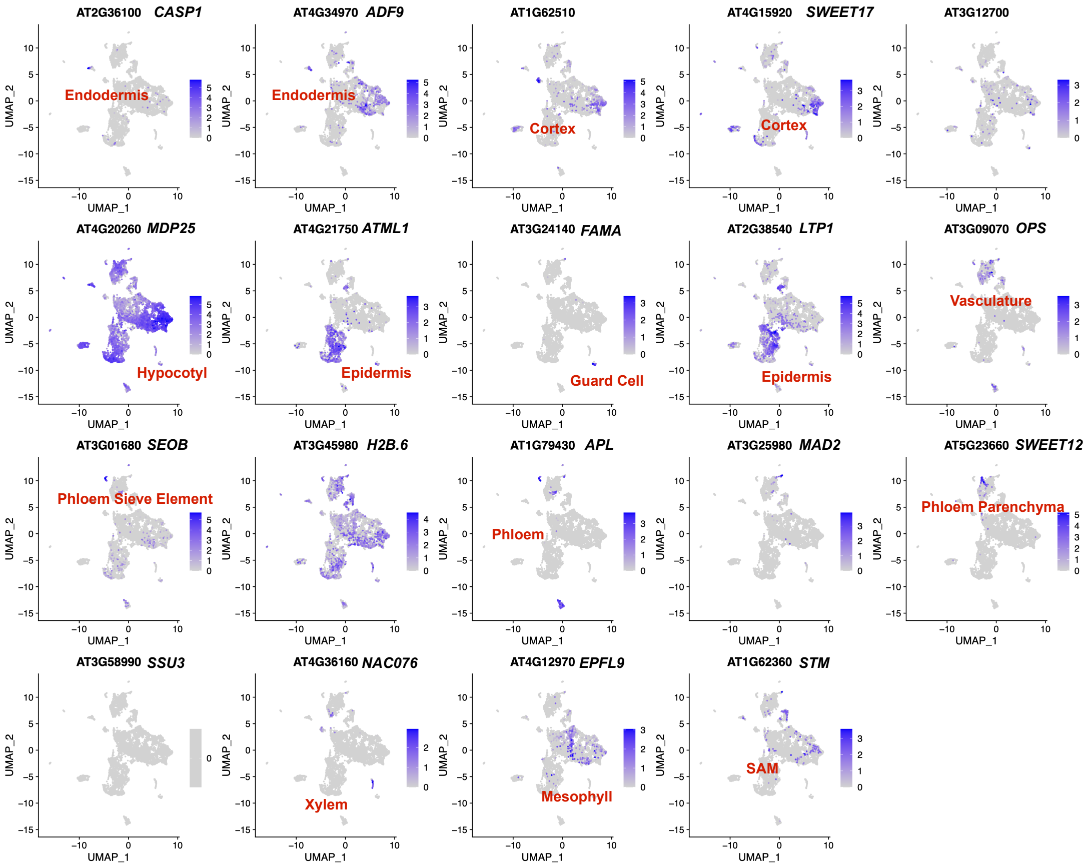

Figure S37 Expression Patterns of Cell Markers on the *pifq* atlas.

Expression of markers for annotation of shoot cell clusters on the atlas constructed for dark-grown seedlings of wild type and the *pifq* mutant.
