# Supplementary material for: Time series single-cell transcriptional atlases reveal cell fate differentiation driven by light in Arabidopsis seedlings
Source: Nat Plants. 2023 Oct 30;9(12):2095–109. doi: 10.1038/s41477-023-01544-4 (PMC10724060; doi:10.1038/s41477-023-01544-4)
Supplement: Supplementary file 2 — Reporting Summary [file 41477_2023_1544_MOESM2_ESM.pdf]

nature portfolio

Corresponding author(s): Hang He; Xing Wang Deng

Last updated by author(s): Aug 9, 2023

Reporting Summary

Nature Portfolio wishes to improve the reproducibility of the work that we publish. This form provides structure for consistency and transparency in reporting. For further information on Nature Portfolio policies, see our Editorial Policies and the Editorial Policy Checklist.

Statistics

For all statistical analyses, confirm that the following items are present in the figure legend, table legend, main text, or Methods section.

n/a	Confirmed	
☐	☒	The exact sample size (*n*) for each experimental group/condition, given as a discrete number and unit of measurement
☐	☒	A statement on whether measurements were taken from distinct samples or whether the same sample was measured repeatedly
☐	☒	The statistical test(s) used AND whether they are one- or two-sided<br>*Only common tests should be described solely by name; describe more complex techniques in the Methods section.*
☐	☒	A description of all covariates tested
☐	☒	A description of any assumptions or corrections, such as tests of normality and adjustment for multiple comparisons
☐	☒	A full description of the statistical parameters including central tendency (e.g. means) or other basic estimates (e.g. regression coefficient) AND variation (e.g. standard deviation) or associated estimates of uncertainty (e.g. confidence intervals)
☒	☐	For null hypothesis testing, the test statistic (e.g. *F*, *t*, *r*) with confidence intervals, effect sizes, degrees of freedom and *P* value noted<br>*Give P values as exact values whenever suitable.*
☐	☒	For Bayesian analysis, information on the choice of priors and Markov chain Monte Carlo settings
☐	☒	For hierarchical and complex designs, identification of the appropriate level for tests and full reporting of outcomes
☐	☒	Estimates of effect sizes (e.g. Cohen's *d*, Pearson's *r*), indicating how they were calculated

Our web collection on statistics for biologists contains articles on many of the points above.

Software and code

Policy information about availability of computer code

Data collection	10x Genomics Chromium Contrller(v3, https://www.10xgenomics.com/instruments/chromium-controller); Illumina Nova-Seq (https://www.illumina.com/systems/sequencing-platforms/novaseq.html); Arabidopsis Information Resource 10 (TAIR10, https://www.arabidopsis.org);
Data analysis	cellranger (v3.0.2, https://support.10xgenomics.com/single-cell-gene-expression/software/pipelines/latest/what-is-cell-ranger); Seurat (v3.20; https://satijalab.org/seurat/); R(v4.02); Hisat2 (v2.2.0, https://ccb.jhu.edu/software/hisat/index.shtml); STRINGTIE (v2.2.1, https://ccb.jhu.edu/software/stringtie/); Trimmomatic (v0.39, www.usadellab.org/cms/?page=trimmomatic); SAMTOOLS (v1.6, https://www.htslib.org); WGCNA (v1.69, https://horvath.genetics.ucla.edu/html/CoexpressionNetwork/Rpackages/WGCNA/); monocle2 (v2.14.0, https://cole-trapnell-lab.github.io/monocle-release/docs/#installing-monocle); Slingshot (v1.8.0, https://bioconductor.org/packages/release/bioc/vignettes/slingshot/inst/doc/vignette.html); ScVelo (v0.2.3, https://scvelo.readthedocs.io/getting_started/); NIS-Elements Viewer (v5.21, https://www.microscope.healthcare.nikon.com/en_EU/products/software/nis-elements/viewer); Perl (v5.32).

For manuscripts utilizing custom algorithms or software that are central to the research but not yet described in published literature, software must be made available to editors and reviewers. We strongly encourage code deposition in a community repository (e.g. GitHub). See the Nature Portfolio guidelines for submitting code & software for further information.

Data

Policy information about availability of data

All manuscripts must include a data availability statement. This statement should provide the following information, where applicable:

- Accession codes, unique identifiers, or web links for publicly available datasets
- A description of any restrictions on data availability
- For clinical datasets or third party data, please ensure that the statement adheres to our policy

All data underlying the findings are available from National Genomics Data Center (PRJCA016521). The gene expression patterns in the de-etiolating atlases could also be obtained from http://182.92.183.62:4576.

Human research participants

Policy information about studies involving human research participants and Sex and Gender in Research.

Reporting on sex and gender	N/A
Population characteristics	N/A
Recruitment	N/A
Ethics oversight	N/A

Note that full information on the approval of the study protocol must also be provided in the manuscript.

Field-specific reporting

Please select the one below that is the best fit for your research. If you are not sure, read the appropriate sections before making your selection.

☒ Life sciences ☐ Behavioural & social sciences ☐ Ecological, evolutionary & environmental sciences

For a reference copy of the document with all sections, see nature.com/documents/nr-reporting-summary-flat.pdf

Life sciences study design

All studies must disclose on these points even when the disclosure is negative.

Sample size	We collected samples large enough in size (≥ 500 seedlings per single cell RNA seq library; >=100 seedlings per bulk RNA seq library with three biological replicates; and >=20 seedlings for per repoterline). Sample size was determined to be adequate based on the magnitude and consistency of measurable differences between different plant lines based on the previous reports.
Data exclusions	No data were excluded from the analysis in the article.
Replication	We collected ≥ 500 seedlings for single cell RNA seq library, comprising 31,796 and 61,065 shoot and root cells which were controls for each other. We sequenced two replicates for dark-grown shoot samples by single cell RNA seq to guarantee the repeatability; We applied three replicates for bulk RNA-seq to guarantee the significance of the difference.
Randomization	The groups in this study are Arabidopsis seedlings grown in different light conditions. The important thing is to posit seedlings in the growth chamber in a way that would ensure as equal growth conditions as possible. e.g. temperature, culture medium. Seedlings grown in different light conditions were posited at random in the growth chamber.
Blinding	Blinding was not applicable to this study because no patient treatment and experiment was applied in this study.

Reporting for specific materials, systems and methods

We require information from authors about some types of materials, experimental systems and methods used in many studies. Here, indicate whether each material, system or method listed is relevant to your study. If you are not sure if a list item applies to your research, read the appropriate section before selecting a response.

Materials & experimental systems

n/a	Involved in the study
☒	☐ Antibodies
☒	☐ Eukaryotic cell lines
☒	☐ Palaeontology and archaeology
☒	☐ Animals and other organisms
☒	☐ Clinical data
☒	☐ Dual use research of concern

Methods

n/a	Involved in the study
☒	☐ ChIP-seq
☒	☐ Flow cytometry
☒	☐ MRI-based neuroimaging
